# The efficacy of baking soda dentifrice in controlling plaque and gingivitis: A systematic review

**DOI:** 10.1111/idh.12390

**Published:** 2019-03-06

**Authors:** Cees Valkenburg, Yasmin Kashmour, Angelique Dao, G. A. (Fridus) Van der Weijden, Dagmar Else Slot

**Affiliations:** ^1^ Department of Periodontology, Academic Centre for Dentistry Amsterdam (ACTA) University of Amsterdam and Vrije Universiteit Amsterdam Amsterdam The Netherlands

**Keywords:** baking soda, bleeding, dentifrice, gingivitis, plaque, sodium bicarbonate, systematic review, toothpaste

## Abstract

**Objective:**

To test the efficacy of a dentifrice containing baking soda (BS), compared with dentifrice without BS for controlling plaque and gingivitis.

**Materials and methods:**

MEDLINE‐PubMed and Cochrane‐CENTRAL were searched. The inclusion criteria were randomized controlled clinical trials including healthy participants aged 18 years or older. Studies were selected that compared the effect of toothbrushing with a dentifrice with and without BS on the clinical parameters of plaque and gingivitis. Data were extracted from the selected studies, and a meta‐analysis was performed.

**Results:**

The search retrieved 21 eligible publications. Among these papers, 43 comparisons were provided, with 23 involving a single‐use design and 20 being evaluations with a follow‐up. Negative controls were found, or positive controls for which various active ingredients had been used. The included studies showed a moderate overall potential risk of bias and considerable heterogeneity. The meta‐analysis of plaque scores from the single‐brushing experiments showed that BS dentifrice (BS‐DF) was associated with significantly better outcomes than the negative control dentifrices (DiffM −0.20; *P* < 0.0001; 95% CI: [−0.27; −0.12]) or the positive control dentifrices (DiffM −0.18; *P* < 0.0001; 95% CI: [−0.24; −0.12]). This finding was only confirmed in studies that used a follow‐up design as compared to a negative control (DiffM −0.19; *P* = 0.01; 95% CI: [−0.34; −0.04]). The indices of gingival bleeding also improved when the comparison was a negative control (DiffM −0.08; *P* = 0.02; 95% CI: [−0.16; −0.01] and (DiffM −0.13; *P* < 0.001; 95% CI: [−0.18; −0.08]. However, for the gingival index scores, the meta‐analysis did not reveal any significant differences.

**Conclusion:**

BS‐DF showed promising results with respect to plaque removal in single‐use studies. However, the finding was partially substantiated in follow‐up studies. Studies that assessed bleeding scores indicated that a small reduction can be expected from BS, relative to a control product.

## 1 INTRODUCTION

Dentifrice, also known as toothpaste, is used in conjunction with a toothbrush to help maintain oral health. The most common components of dentifrice are an abrasive agent, a binder, a surfactant and a humectant. The main intention of the use of paste is to help remove debris and plaque but it also has secondary functions such as breath freshening and tooth whitening, which are widely marketed. There is an almost universal recommendation that people should brush their teeth twice a day with a fluoridated dentifrice.^1^

Several dentifrice manufacturers have incorporated sodium bicarbonate, commonly known as baking soda (BS), into their formulas. This is a salt composed of sodium ions and bicarbonate ions. BS is nontoxic and is mild on the soft tissues of the gums and oral mucosa. In commercial dentifrices, BS mainly serves the purpose of an abrasive. Relative dentin abrasion tests have shown that the abrasiveness of sodium bicarbonate has low abrasivity of the tooth surface. It is an alkaline substance capable of neutralizing acids. As such, it potentially can prevent tooth decay by neutralizing the acids produced by bacteria in the mouth.^2^ BS also neutralizes acidic components of common tooth‐staining chemicals, such as the chromogens in tea, and red wine,^3^ thereby lessening their staining potential.

The current widespread use of BS in dentifrices and home oral hygiene regimens is largely attributable to the impact of Dr Paul H. Keyes.^4^ In the 1970s, he was among the first to employ anti‐infective agents and microbiological testing in non‐surgical periodontal therapy, including patient home irrigation with BS or salt solutions, and brushing with a mix of BS and hydrogen peroxide. This approach is known as “the Keyes technique,” popularly referred to as the “salt‐and‐soda” method. The method became widely integrated into people's oral hygiene routines. However, it was critically evaluated by the American Academy of Periodontology from which it was concluded that the benefits of the technique are almost exclusively derived from the detailed oral hygiene procedures and root planning.^4^

Nowadays, BS is found in many dentifrices. In an era with upcoming preference for “assumed” naturally based products,^5^ it is important to investigate the associated oral health benefits. Until this study, no systematic evaluation had been conducted on the adjuvant effect of sodium bicarbonate in dentifrices. The aim of this systematic review (SR) was to establish the effect of BS on plaque removal and gingivitis.

## 2 MATERIALS AND METHODS

This SR was prepared and described in accordance with the Cochrane handbook for systematic reviews of interventions^6^ and the guidelines in Transparent Reporting of Systematic Reviews and Meta‐analysis (PRISMA‐statement).^7^ The protocol for this review was developed “a priori” and registered with the International Prospective Register of Systematic Reviews^8^ under the registration number CRD42018080649. All post hoc changes were appropriately noted (see Appendix S1).

### 2.1 Focused question

In healthy individuals, what is the efficacy of toothbrushing with a dentifrice that contains BS compared to a dentifrice without BS on clinical indices of plaque and gingivitis?

### 2.2 Search strategy

A structured search strategy was designed to retrieve all relevant studies. As proposed in the Cochrane handbook, the National Library of Medicine, Washington, DC (MEDLINE‐PubMed) and the Cochrane Central Register of Controlled Trials (CENTRAL) were searched from initiation to September 2018 for papers related to the focused research question. The reference lists of the included studies were hand‐searched to identify additional potentially relevant studies. No limitations were placed on language or date of publication in the electronic searches of the databases. For details regarding the search terms used, see Table 1.

**Table 1:**
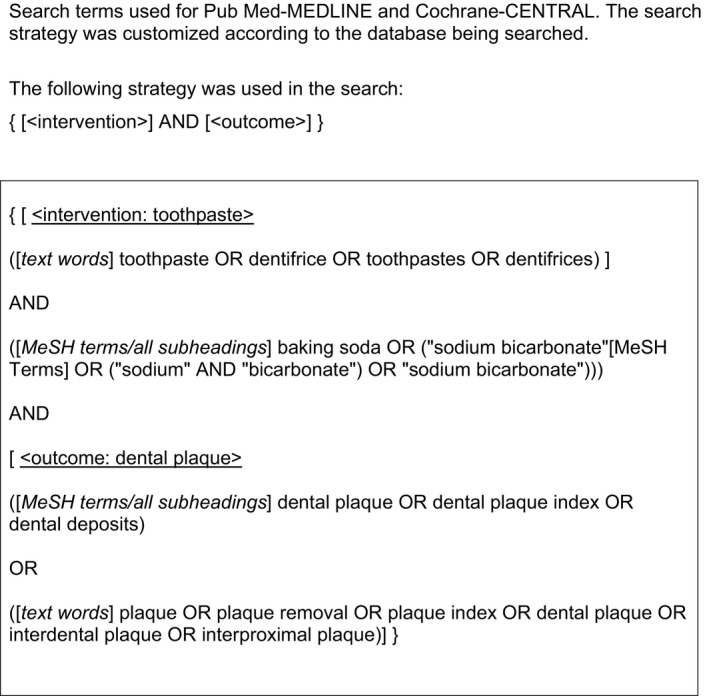
Search strategy

### 2.3 Screening and selection

The titles and abstracts of the studies obtained from the searches were screened independently by three reviewers (AD, YK and CV) to select studies that potentially met the inclusion criteria. No language restrictions were imposed. Based on the title and abstract, the full‐text versions of potentially relevant papers were obtained. These papers were categorized (by CV and DES) as definitely eligible, definitely not eligible or questionable. Disagreements concerning eligibility were resolved by consensus, and if disagreement persisted, the decision was resolved through arbitration by another reviewer (GAW). Papers that fulfilled all the inclusion criteria were processed for data extraction.

The included full report studies were considered to meet the following criteria: (a) the study design was either a randomized controlled clinical trial (RCT) or a controlled clinical trial (CCT), (b) the studies were conducted with healthy participants, who were not institutionalized and were 18 years of age or older, (c) the studies included participants without orthodontic treatment and/or removable prostheses, (d) as an intervention, a dentifrice with BS was evaluated in comparison with a dentifrice without this ingredient, (e) chlorhexidine was not an ingredient incorporated in a dentifrice, (f) rinsing with an additional antiseptic was not a part of the intervention or control regimen and (g) the studies evaluated plaque and gingivitis scores. For details, see Appendix S2.

### 2.4 Assessment of heterogeneity

The following factors were used to evaluate the heterogeneity of the outcomes of the different studies: study design, participant characteristics, study group details and regimens. In addition, side effects and industry funding were evaluated.

### 2.5 Assessment of methodological quality and risk of bias

All included studies were independently scored for their methodological quality by three reviewers (AD, CV and YK). Disagreement was resolved by consensus, and if disagreement persisted, the decision was resolved through arbitration by a fourth reviewer (DES). The assessed items are detailed in Appendix S3.^9^

### 2.6 Data extraction

The characteristics of the population, intervention, comparison and outcomes were extracted from all studies independently by two reviewers (AD and YK) using a specially designed data extraction form. A third reviewer (CV) also read the full texts of the included trials and, independently from the two others, checked the data extracted. Disagreement between the reviewers was resolved through discussion and consensus. If this was not satisfactory, the judgement of another reviewer (GAW) was decisive. Means and standard deviations (SDs) were extracted. Some studies provided standard errors (SEs) of the means. Where possible, the current authors calculated SD based on the sample size (SE = SD/√N). For those papers that provided insufficient data to be included in the analysis, the first and/or corresponding author was contacted to request additional data.

### 2.7 Data analysis

Studies were categorized as single‐brushing designs that were selected to evaluate a change in plaque scores. Studies with a follow‐up were selected to evaluate plaque as well as gingivitis scores. The dentifrices without BS were separated into negative and positive controls. As a positive control, dentifrices containing stannous fluoride (SnF) or triclosan (Tcs) as ingredients were considered.^10^, ^11^ All the other dentifrices without BS were considered as negative controls. As a summary, a descriptive data presentation was used for all studies.

Where feasible, a meta‐analysis (MA) was performed with at least two included experiments evaluating the same outcome parameter. When a study had multiple non‐BS dentifrice treatment arms, and data from the BS‐DF were used in more than one comparison, the number of participants (n) in that group was divided by the number of comparisons. The difference of means (DiffM) between the test and control groups was calculated using a “random effects” model with an “inverse variance” method as proposed by DerSimonian and Laird.^12^ The primary method of calculating all pooled estimates and a sub‐analysis was performed with the Knapp‐Hartung adjustment^13^ in cases of at least five eligible studies.^14^ For meta‐analyses with more than two comparisons, 95% predictive intervals were calculated to quantify treatment effects in a future clinical setting.^15^

Heterogeneity was tested using the chi‐square test and the *I* ^2^ statistic. A chi‐squared test resulting in *P* < 0.1 was considered to be an indication of significant statistical heterogeneity. If possible, the formal testing for publication bias using the minimum amount of 10 comparisons was applied, as proposed by Egger et al^16^ and Sterne et al.^17^

A sub‐analysis was performed using a network meta‐analysis (NMA).^18^, ^19^, ^20^, ^21^, ^22^ Treatments were ranked^23^, ^24^ through a frequentist weighted least squares approach, as described by Rücker.^25^, ^26^ The direct evidence proportion as described in König et al^27^ was used to calculate the indirect evidence.^26^ A decomposition of heterogeneity within designs and between designs was provided,^28^ and a net heat plot graphical tool, as proposed by Krahn et al,^29^ was used to locate inconsistency in the NMA.^29^ For the transitivity assumption,^11^, ^30^ the ingredients were analysed. All computations were performed using R (https://www.r-project.org) with the packages meta,^31^ metafor^32^ and netmeta.^26^

### 2.8 Grading the “body of evidence”

The Grading of Recommendations Assessment, Development and Evaluation (GRADE) system was used to rank the evidence.^33^ Two reviewers (CV and DES) rated the quality of the evidence and the strength and direction of the recommendations^34^ according to the following aspects: risk of bias, consistency of results, directness of evidence, precision and publication bias and magnitude of the effect. Any disagreement between the two reviewers was resolved after additional discussion with a third reviewer (GAW).

## 3 RESULTS

### 3.1 Search and selection results

**Figure 1:**
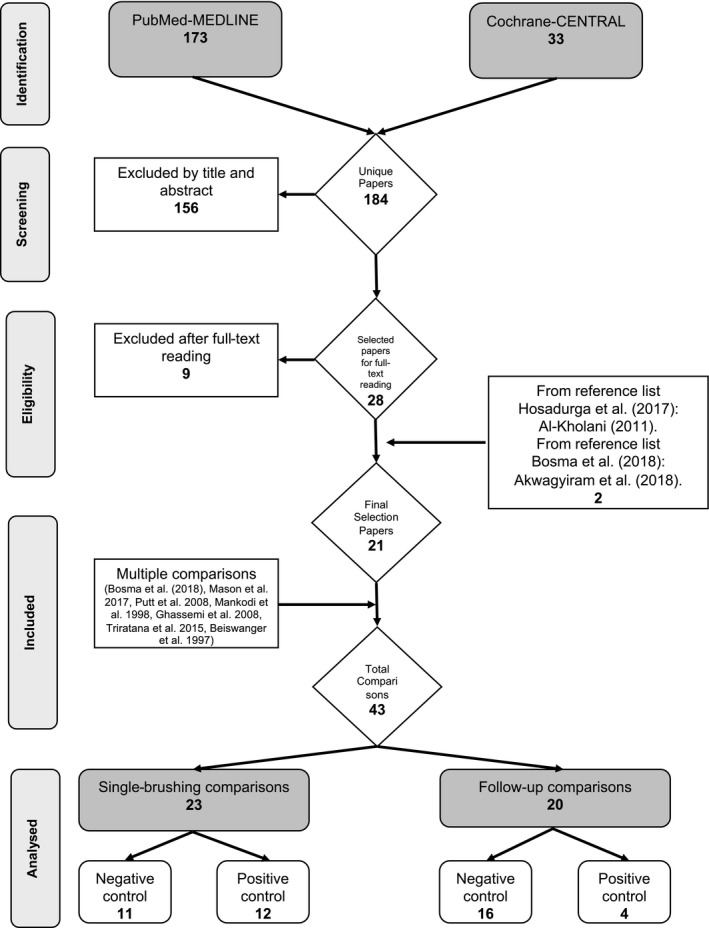
Search and selection results

The search of the MEDLINE‐PubMed and Cochrane‐CENTRAL databases resulted in 184 unique papers. Manual searching of the reference lists of the final selected papers provided two additional relevant papers, Al‐Kholani et al,^35^ listed by Hosadurga et al^36^ and Akwagyiram et al,^37^ listed by Bosma et al.^38^ Altogether, 21 eligible publications were found.^5^, ^35^, ^36^, ^37^, ^38^, ^39^, ^40^, ^41^, ^42^, ^43^, ^44^, ^45^, ^46^, ^47^, ^48^, ^49^, ^50^, ^51^, ^52^, ^53^, ^54^ Among these, Putt et al^49^ and Mason et al^53^ provided five and two sub‐studies, respectively, within their main publications. Ghassemi et al^50^ provided within one study model, two single‐brushing exercises and also one study with a follow‐up. Finally, 43 comparisons were identified. A single‐brushing design was used in 23 comparisons, 12 of which had a positive control and 11 with a negative control. For the 20 brushing comparisons with a follow‐up, 16 had a negative control and 4 had a positive control. For negative controls, sodium fluoride (NaF), monofluorophosphate (MFP) and any other dentifrices without BS (non‐BS) were considered. For details, see Figure 1.

### 3.2 Heterogeneity

The included studies exhibited considerable heterogeneity with respect to the study design, participant characteristics, study group details and the regimens used. Information regarding the study outline and characteristics is shown in detail in Appendix S2.

Studies used different indices^55^, ^56^ and values for plaque and gingivitis as the inclusion criteria. Smoking status was generally unclear. Hosadurga et al^36^ specifically excluded smokers, and only Lomax et al,^52^ Jose et al^54^ and Akwagyiram et al^37^ reported the smoking status of the included participants. The duration of follow‐up studies ranged from 1 month to 6 months. In five studies,^5^, ^35^, ^37^, ^46^, ^52^ participants received professional oral prophylaxis at the start of the experimental period. The RDA value, the percentage of BS and the fluoride content were inconsistently reported. Most studies also did not report the average brushing time. Seven studies mentioned a brushing time of 1 minute, of which three included single‐brushing exercises with supervised 1‐minute brushing.^37^, ^38^, ^47^, ^49^, ^53^, ^54^ The majority of the studies provided their participants with a standard toothbrush, but two studies, Yankell et al^42^ and Yankell and Emling,^40^ allowed the participants to use their own toothbrush. However, all these factors could not be further analysed in the current review.

### 3.3 Adverse effects

Twelve papers^5^, ^36^, ^37^, ^38^, ^44^, ^45^, ^48^, ^50^ mentioned evaluation for possible adverse effects. Only in one study did four participants discontinue the study because of disliking the dentifrice taste.^45^ In another study, the participants complained of an unpleasant taste in the initial period when using a BS‐DF.^36^ Ulcerations were reported in one study, but they appeared unrelated to the trial and eventually.^5^ In Winer et al,^39^ two persons were dropped out of the experimental group, which was suggested to be product‐related. In one study, a participant experienced a mild burning sensation and moderate dental hypersensitivity.^54^

### 3.4 Industry funding

Most of the 21 included studies reported on the use of commercially available dentifrices and toothbrushes. For three studies, it was unclear whether the dentifrices were marketed products.^35^, ^39^, ^40^ Five other studies used non‐marketed experimental dentifrices.^38^, ^42^, ^45^, ^52^, ^54^ Fifteen studies had industry involvement, with seven different companies acting either as a study initiator or where the authors were employees; companies also provided products, funding or financial grants. Five studies did not mention industry connections and one study included a disclosure statement of no financial interest.^39^

### 3.5 Methodological quality and assessment of bias

To estimate the potential risk of bias, the methodological qualities of the included studies were used, as assessed in the checklist presented in Appendix S3 (methodological quality and potential risk of bias scores of the individual included studies). Based on a summary of the proposed criteria, the estimated potential risk of bias was low for nine studies,^5^, ^37^, ^38^, ^45^, ^48^, ^51^, ^53^, ^54^ moderate for six studies^42^, ^43^, ^44^, ^47^, ^50^ and high for six studies.^35^, ^39^, ^40^, ^41^, ^46^

### 3.6 Study outcome results

Appendix S4 presents the results of the data extraction. Baseline scores, end scores and incremental changes within each intervention group are presented.

### 3.7 Descriptive analysis

Table 2 provides a descriptive summary of the significant differences between toothbrushing with a BS‐DF and without BS as reported by the original authors. In all but one of the 23 comparisons that presented results using the single‐brushing design, when BS‐DF was compared to either a negative control or a positive control, it was found to be significantly more effective for plaque removal (Table 2A).

**Table 2:** A descriptive summary of the statistical significance of individual study outcomes for the single‐brushing and long‐term studies. (A) Descriptive summary of the single‐brushing dentifrice comparisons; (B) Descriptive summary follow‐up dentifrice comparisons

(A)				
Control	Study (year)	% BS	Plaque score	Comparison
Negative	Bosma et al (2018) A^38^	67	>	NaF
	Bosma et al (2018) B^38^	67	>	NaF
	Bosma et al (2018) C^38^	62	>	NaF
	Mason et al (2017) 1A^53^	45	>	NaF
	Mason et al (2017) 1B^53^	67	>	NaF
	Putt et al (2008) 3A^49^	27	>	NaF
	Putt et al (2008) 3B^49^	48	>	NaF
	Putt et al (2008) 4^49^	65	>	NaF
	Emling and Yankell (1988)^41^	?	=	NaF
	Mankodi et al (1998) B^47^	65	>	NaF
	Mankodi et al (1998) C^47^	65	>	MFP+NaF
Total			10/11>	
Positive	Ghassemi et al (2008) 1^50^	?	>	Tcs
	Ghassemi et al (2008) 2^50^	?	>	Tcs
	Putt et al (2008) 1A^49^	20	>	Tcs
	Putt et al (2008) 1B^49^	65	>	Tcs
	Putt et al (2008) 2A^49^	20	>	Tcs
	Putt et al (2008) 2B^49^	48	>	Tcs
	Putt et al (2008) 3A^49^	27	>	Tcs
	Putt et al (2008) 3B^49^	48	>	Tcs
	Putt et al (2008) 5^49^	20	>	Tcs
	Mason et al (2017) 2^53^	67	>	SnF
	Putt et al (2008) 2A^49^	20	>	SnF
	Putt et al (2008) 2B^49^	48	>	SnF
Total			12/12>	

(B)						
Control	Study (year)	% BS	Plaque score	Gingival Index	Bleeding score	Comparison
Negative	Akwagyiram et al (2018)^37^	67	>	>	>*	NaF
	Jose et al (2018)^54^	67	>	>	>*	NaF
	Lomax et al (2016)^52^	67	□	>	>*	NaF
	Al‐Kholani et al (2011)^35^	?	>	>	>	NaF
	Yankell and Emling (1988)^40^	?	□	=	>*	NaF
	Beiswanger et al (1997)^46^	?	=	=	=	NaF
	Hosadurga et al (2017)^36^	?	=	=	□	MFP
	Triratana et al (2015)^51^	?	=	=	□	MFP+NaF
	Mullally et al (1995)^5^	?	=	=	=	MFP+NaF
	Al‐Kholani et al (2011)^35^	?	=	=	=	Non‐BS
	Saxer et al (1995)^45^	?	=	=	=	Non‐BS
	Saxer et al (1994)^44^	?	□	□	>*	Non‐BS
	Taller (1993)^43^	?	□	□	=	Non‐BS
	Yankell et al (1993)^42^	?	>	=	>*	Non‐BS
	Yankell and Emling (1988)^40^	?	?	>	>*	Non‐BS
	Winer et al (1986)^39^	?	=	>	□	Non‐BS
Total			7/16=;4/16>	8/16=;6/16>	5/16=;8/16>	
Positive	Triratana et al (2015)^51^	?	>	=	□	Tcs
	Ghassemi et al (2008)^50^	?	>	□	□	Tcs
	Ozaki et al (2006)^48^	?	=	=	□	Tcs
	Beiswanger et al (1997)^46^	?	=	<	<	SnF
Total			2/4=;2/4>	2/4=	NA	

>: significant difference in favour of the BS‐DF group, <: significant difference in favour of the control group, =: no significant difference, □: no data available, *: multiple indices, NA: not applicable, % BS: percentage baking soda in dentifrice.

Plaque index scores obtained from the majority of studies with a follow‐up showed a pattern of no difference between BS‐DF and their controls. An inconsistent pattern was noted for the gingival index scores and for bleeding scores when a negative control was used. In two of the three comparisons that used Tcs‐DF as a positive control, BS‐DF showed a significant improvement in plaque removal. This could not be confirmed with respect to gingival health scores. The only study that used SnF‐DF as a positive control showed that it was significantly more effective than BS‐DF regarding the gingival index and bleeding scores (Table 2B).

### 3.8 Meta‐analysis

The obtained data allowed for several meta‐analyses, which could be performed separately for plaque, gingivitis and bleeding index scores. The results are summarized in Table 3. The forest plots and corresponding funnel plots that illustrate these outcomes are shown in Appendix S6‐S12. For the studies that evaluated a single‐use design, a meta‐analysis based on the Turesky et al (1970) modification of the Quigley and Hein Plaque Index (1962) (TQ&H) was feasible. Compared to a negative control DF, the difference in means for end scores (−0.20; *P* < 0.0001; 95% CI: [−0.27; −0.12]) and incremental scores (−0.21; *P* < 0.0001; 95% CI: [−0.27; −0.16]) showed a significant effect in favour of BS. A similar finding was present when the control was a positive control DF for end scores (DiffM −0.18; *P* < 0.0001; 95% CI: [−0.24; −0.12]) and incremental scores (DiffM −0.18; *P* < 0.0001; 95% CI: [−0.22; −0.14]). These findings are supported by the corresponding prediction intervals.

**Table 3:** Overview of the meta‐analysis of the reported indices included in this systematic review. (A) Single‐brushing design: Meta‐analysis for single‐brushing design studies for the Turesky et al (1970) modification of the Quigley & Hein (1962) plaque Index. The baseline, end and difference data evaluating a dentifrice with BS compared to a negative either a positive control dentifrice; (B) Follow‐up studies compared to a negative or positive control dentifrice. (B1) Meta‐analysis for follow‐up brushing design studies for the baseline, end and difference data evaluating a dentifrice with BS compared to a negative either a positive control dentifrice according to plaque indices; (B2) Meta‐analysis for follow‐up brushing design studies for the baseline, end and difference data evaluating a dentifrice with BS compared to a negative either a positive control dentifrice according to GINGIVAL Index; (B3) Meta‐analysis for follow‐up brushing design studies for the baseline, end and difference data evaluating a dentifrice with BS compared to a negative control dentifrice according to BLEEDING indices; (C) Sub‐meta‐analysis on ingredients for follow‐up brushing design dentifrice comparisons end data evaluating a dentifrice with BS compared to a negative control dentifrice. plaque indices for the Turesky et al (1970) modification of the Quigley & Hein (1962) and the Silness & Löe (Silness & Löe 1964) including the modification by Löe (1967)

(A)									
Single‐brushing design		Comparison	Effect size			Heterogeneity		Prediction Interval (≥3 comparisons)	For details see appendix
Control	Included studies		DiffM	95% CI	*P*‐value	*I* ^2^ 95% CI	*P*‐value		
Negative control	**Negative control** 9 comparisons Emling & Yankell (1988)^41^ Putt et al (2008)* 49 3x Mason et al (2017)* 53 2x Bosma et al (2018)* 38 3x	Baseline	0.01	(−0.05; 0.07)	0.67	0% (0%‐0%)	1.00	(−0.06; 0.09)	S6a1
		End	−0.20	(−0.27; −0.12)	**<0.0001**	0% (0%‐38%)	0.80	(−0.28; −0.11)	S6a3
	**Negative control** 8 comparisons Putt et al (2008)* 49 3x Mason et al (2017)* 53 2x Bosma et al (2018)* 38 3x	Difference	−0.21	−0.27; −0.16	**<0.0001**	0% (0%‐57%)	0.62	(−0.28; −0.14)	S6a5
Positive control	**Positive control** 12 comparisons Putt et al (2008)* 49 x Ghassemi et al (2008)* 50 2x Mason et al (2017)^53^	Baseline	−0.01	(−0.06; 0.04)	0.66	0% (0%‐22%)	0.88	(−0.06; 0.04)	S6a2 Funnel plot S9A
		End	−0.18	(−0.24; −0.12)	**<0.0001**	30% (0%‐65%)	0.15	(−0.33; −0.03)	S6a4 Funnel plot S9B
		Difference	−0.18	(−0.22; −0.14)	**<0.0001**	27% (0%‐63%)	0.18	(−0.27; −0.09)	S6a6

(B1)									
Follow‐up brushing design compared to negative and positive control		Comparison	Effect size			Heterogeneity		Prediction Interval (≥3 comparisons)	For details see appendix
Index	Included studies		DiffM	95% CI	*P*‐value	*I* ^2^ 95% CI	*P*‐value		
PI TQ&H^a^	**Negative control** 9 comparisons Yankell & Emling (1988)*, 40 2x Yankell et al (1993)^42^ Mullally et al (1995)^5^ Saxer et al (1995)^45^ Triratana et al (2015)^51^ Hosadurga et al (2017)^36^ Jose et al (2018)^54^ Akwagyiram et al (2018)^37^	Baseline	0.01	(−0.03; 0.05)	0.59	0% (0%‐60%)	0.53	(−0.04; 0.06)	S6‐B1
		End	−0.19	(−0.34; −0.04)	**0.01**	85% (72%‐91%)	**<0.01**	(−0.69; 0.31)	S6‐B3
	**Negative control** 3 comparisons Mullally et al (1995)^5^ Triratana et al (2015)^51^ Hosadurga al. (2017)^36^	Difference	0.02	(−0.20; 0.24)	0.84	74% (12%‐92%)	0.17	(−2.51; 2.55)	S6‐B5
	**Positive control** 3 comparisons Ozaki et al (2006)^48^ Ghassemi et al (2008)^50^ Triratana et al (2015)^51^	Baseline	−0.09	(−0.09; 0.09)	0.94	0% (0%‐0%)	0.99	(−0.58; 0.59)	S6‐B2
		End	0.44	(−0.80; 1.69)	0.49	99% (99%‐99%)	**<0.01**	(−15.65; 16.53)	S6‐B4
		Difference	0.42	(−0.39; 1.24)	0.31	99% (98%‐99%)	**<0.01**	(−10.09; 10.94)	S6‐B6
PI S&L^b^	**Negative control** 2 comparisons Winer et al (1986)^39^ Beiswanger et al (1997)^46^	Baseline	0.06	(−0.02; 0.14)	0.14	0%	0.76	NA	S6‐C1
		End	0.04	(−0.01; 0.10)	0.14	0%	0.67	NA	S6‐C2

(B2)									
Follow‐up brushing design compared to negative and positive control		Comparison	Effect size			Heterogeneity		Prediction Interval (≥3 comparisons)	For details see appendix
Index	Included studies		DiffM	95% CI	*P*‐value	*I* ^2^ 95% CI	*P‐*value		
GI L&S^c^	**Negative control** 11 comparisons Winer et al (1986)^39^ Yankell and Emling (1988)*, 40 2x Yankell et al (1993)^42^ Mullally et al (1995)^5^ Saxer et al (1995)^45^ Beiswanger et al (1997)^46^ Al‐Kholani et al (2011)* 2x^35^ Triratana et al (2015)^51^ Hosadurga et al (2017)^36^	Baseline	0.02	(−0.01;0.04)	0.23	0.0% (0%‐52%)	0.61	(−0.01; 0.04)	S7‐1
		End	−0.04	(−0.11; 0.03)	0.25	71% (45%‐84%)	**<0.01**	(−0.24; 0.16)	S7‐3
	**Negative control** 3 comparisons Mullally et al (1995)^5^ Triratana et al (2015)^51^ Hosadurga et al (2017)^36^	Difference	−0.07	(−0.14; 0.00)	**0.04**	0% (0%‐10%)	0.68	(−0.51; 0.37)	S7‐5
	**Positive control** 3 comparisons Beiswanger et al (1997)^46^ Ozaki et al (2006)^48^ Triratana et al (2015)^51^	Baseline	0.01	(−0.04; 0.07)	0.59	0% (0%‐60%)	0.77	(−0.33; 0.36)	S7‐2
		End	0.24	(−0.04; 0.51)	0.09	93% (83%‐97%)	**<0.01**	(−3.21; 3.69)	S7‐4
	**Positive control** 2 comparisons Ozaki et al (2006)^48^ Triratana et al (2015)^51^	Difference	0.31	(−0.11; 0.73)	0.15	95%	**<0.01**	NA	S7‐6
MGI^f^	**Negative control** 3 comparisons Akwagyiram et al (2018)^37^ Jose et al (2018)^54^ Lomax et al (2016)^52^	Baseline	−0.01	(−0.05; 0.03)	0.54	0% (0%‐16%)	0.88	(−0.26; 0.23)	S7‐7A
		End	−0.37	(−0.61; −0.14)	**<0.001**	96% (91%‐98%)	**<0.01**	(−3.41; 2.66)	S7‐7B

(B3)									
Follow‐up brushing design compared to negative control		Comparison	Effect size			Heterogeneity		Prediction Interval (≥3 comparisons)	For details see appendix
Index	Included studies		DiffM	95% CI	*P*‐value	*I* ^2^ 95% CI	*P*‐value		
BI GBI^d^	**Negative control** 4 comparisons Yankell and Emling (1988)* 2x^40^ Yankell et al (1993)^42^ Taller (1993)^43^	Baseline	0.03	(−0.06; 0.12)	0.56	54% (0%‐85%)	0.09	(−0.32; 0.37)	S8‐A1
		End	−0.08	(−0.16; −0.01)	**0.02**	14% (0%‐87%)	0.32	(−0.28; 0.11)	S8‐A2
BI Saxer^e^	**Negative control** 5 comparisons Saxer et al (1994)^44^ Saxer et al (1995)^45^ Lomax et al (2016)^52^ Jose et al (2018)**, 54 Akwagyiram et al (2018)^37^	Baseline	0.00	(−0.02; 0.03)	0.84	2% (0%‐80%)	0.39	(−0.05; 0.05)	S8‐B1
		End	−0.13	(−0.18; −0.08)	**<0.001**	69% (21%‐88%)	**0.01**	(−0.29; 0.03)	S8‐B2

(C)									
Follow‐up brushing design compared to negative control		Included studies	Effect size			Heterogeneity		Prediction Interval (≥3 comparisons)	For details see appendix
Index	Ingredient		DiffM	95% CI	*P*‐value	*I* ^2^ 95% CI	*P*‐value		
PI TQ&H	NaF	3 comparisons: Akwagyiram et al (2018)^37^ Jose et al (2018)^54^ Yankell and Emling B 1988^40^	−0.43	(−0.49; −0.36)	**<0.0001**	33% (0%; 78%)	0.22	(−1.08; 0.23)	S6‐B3
	MFP	3 comparisons: Mullally (1995)^5^ Triratana A (2015)^51^ Hosadurga (2017)^36^	−0.07	(−0.29; 0.14)	0.50	77% (24%; 93%)	**0.01**	(−2.64; 2.49)	
	Non‐BS	3 comparisons: Yankell and Emling A (1988)^40^ Yankell (1993)^42^ Saxer (1995)^45^	−0.13	(−0.37; 0.11)	0.28	51% (0%; 86%)	0.13	(−2.57; 2.30)	
GI L & S	NaF	3 comparisons: Yankell and Emling B (1988)^40^ Beiswanger A (1997)^46^ Al‐Kholani B (2011)^35^	−0.10	(−0.26; 0.07)	0.26	55% (0%; 87%)	0.11	(−1.85; 1.66)	S7‐3
	MFP	3 comparisons: Mullally (1995)^5^ Hosadurga (2017)^36^ Triratana A (2015)^51^	−0.08	(−0.15; −0.02)	**0.01**	0% (0%; 69%)	0.72	(−0.49; 0.33)	
	Non‐BS	5 comparisons: Winer (1986)^39^ Yankell and Emling A (1988)^40^ Yankell (1993)^42^ Saxer (1995)^45^ Al‐Kholani A (2011)^35^	0.07	(0.04; 0.10)	**<0.0001**	0% (0%; 68%)	0.63	(0.02; 0.12)	

Note

NA: not applicable. *P*‐values are presented in bold if *P* ≤ 0.05.

a The Turesky et al (1970)^60^ modification of the Quigley and Hein Plaque Index (1962).^55^

b The Löe (1967)^58^ modification of the Silness & Löe Plaque Index (1964).^57^

c The Löe & Silness Gingival Index (1963)^56^ and The Löe & Silness Gingival Index (1967).^58^

d The Ainamo & Bay Gingival Bleeding Index (1975)^62^ and The Abrams, Caton and Polson Bleeding on Probing Index (1984).^65^

e The Saxer et al (1977)^64^ Papillary Bleeding Index modification of the Ainamo & Bay Bleeding Index (1975)^62^ and The Saxton & Van der Ouderaa (1989) Gingival Bleeding Index.^68^

f The Lobene et al (1986) modification of the Gingival Index (MGI).^69^

* Multiple comparisons with the number taken from this publication.

** Only end scores.

The studies using a follow‐up design evaluated plaque scores on the TQ&H to compare the treatment group to negative and positive controls. The comparison with negative controls showed a significant effect (−0.19; *P* = 0.01; 95% CI: [−0.34; −0.04]) but not when the prediction interval was considered. The Löe (1967) modification of the Silness & Löe Plaque Index (S&L) was used in studies with a negative control. None showed a significant effect. A similar pattern was noted for the Löe & Silness Gingival Index (1963) and the Löe & Silness Gingival Index (1967) (L&S). No significant difference was found for either the comparison with a negative control or the comparison with a positive control. Also, no significant difference was found in the incremental scores, when the 95% prediction interval was considered.

Analysis of bleeding scores was possible only for comparisons with a negative control DF. Using the Saxer et al (1977) Papillary Bleeding Index modification of the Ainamo & Bay Bleeding Index (1975) and the Saxton & van der Ouderaa (1989) Gingival Bleeding Index, a significant difference in means was found for end scores (DiffM −0.13; *P* < 0.001; 95% CI: [−0.18; −0.08]). The 95% prediction interval included the null or opposite direction [−0.29; 0.03]. When the Ainamo & Bay Gingival Bleeding Index (1975) and the Abrahams, Caton and Polson Bleeding on Probing Index (1984) were used, the end scores indicated a significant effect (DiffM −0.08; *P* < 0.02; 95% CI: [−0.16; −0.01]), again with a 95% prediction interval including the null or opposite direction [−0.28; 0.11].

The publication bias was formally tested as indicated. Contour‐enhanced funnel plots^70^, ^71^ showing 10 or more comparisons are presented in Appendix S9‐S10. The asymmetric shape of the funnel plot and the Egger's test of the follow‐up brushing exercises analysing end gingival scores of the Löe & Silness (1963) Gingival Index suggest that the presence of publication bias is likely.

The findings of the MA were supported by the NMA when the heterogeneity and the inconsistency across networks were accounted for. For details of the NMA results, see Appendix S11.

## 4 EVIDENCE PROFILE

Table 4 presents a summary of the various factors used to rate the quality of evidence and to appraise the strength and direction of recommendations according to GRADE.^33^ There is evidence from single‐brushing studies to support the use of BS as an ingredient for improving plaque removal. However, because of the fact that this design does not replicate home use, it is considered indirect evidence. With a moderate precision, the strength and direction of the recommendation based on single‐use studies were estimated to be “weakly in favour.”

**Table 4:** Summary of findings table on body of the estimated evidence profile (Guyatt et al, 2008) and appraisal of the strength of the recommendation regarding the efficacy of BS as ingredient added to a dentifrice on the parameters of interest

Study design	Plaque				Bleeding		Gingivitis	
	Single‐brushing		Follow‐up		Follow‐up		Follow‐up	
	Negative control	Positive control	Negative control	Positive control	Negative control	Positive control	Negative control	Positive control
# Comparisons descriptive analysis (Figure 1, Table 2)	8	12	10	4	11	1	12	3
# Comparisons in meta‐analysis (Table 3)	4	11	7 + 2	3	4 + 3	NA	11	3
Risk of bias (Online Appendix S3)	Low‐high	Low‐high	Low‐high	Low‐high	Low‐high	high	Low‐high	Low‐high
Consistency	Consistent	Consistent	Inconsistent	Inconsistent	Rather consistent	NA	Inconsistent	Inconsistent
Directness	Slightly	Indirect	Direct	Direct	Direct	NA	Direct	Direct
Precision	Rather precise	Precise	Precise	Rather precise	Rather precise	NA	Precise	Rather precise
Reporting bias	Possible	Possible	Possible	Possible	Possible	NA	Possible	Possible
Magnitude of the effect (Table 3)	Small	Small	No difference	No difference	No difference	NA	No difference	No difference
Strength and direction of the recommendation based on the quality and body of evidence	Weak in favour of	Weak in favour of	Moderate certainty of no difference	Moderate certainty of no difference	Moderate certainty of no difference	NA	Moderate certainty of no difference	Moderate certainty of no difference
Recommendation	With respect to plaque and gingivitis, BS dentifrice may be considered as an alternative for other commercially available dentifrices.							

No difference was determined for plaque scores and gingivitis index scores in studies with a follow‐up. However, based on the statistically significant difference in means and the prediction intervals, in future studies, a small difference in bleeding scores between controls and experimental participants can be expected. Given the strength of the recommendation, there is a “moderate” certainty that the BS‐DF did not provide an additional benefit in the studies with a follow‐up. The efficacy of BS‐DF is comparable to that of other commercially available dentifrices.

## 5 DISCUSSION

From a previously published meta‐review on the evidence for dentifrices, it appeared that there was a lack of a systematic appraisal of the evidence concerning the efficacy of BS‐DF.^74^ Therefore, the aim of this systematic review was to assess the effect of toothbrushing with a BS‐DF on plaque and the clinical parameters of gingivitis. Data were extracted from 21 studies which included 2517 participants. The present SR shows, based on the single‐brushing experiments, a small but significant improvement of plaque removal when toothbrushing is performed with a BS dentifrice. However, no favourable effect of BS on plaque scores was found in studies with a follow‐up when the prediction interval was considered. On the other hand, follow‐up studies have shown that on bleeding scores a small effect with a 95% prediction interval including the null or opposite direction can be expected from the use of BS.

The MA in this review differentiated between single‐use brushing exercises and the longer‐term effect of brushing, in order to eliminate design‐related differences. Also, it distinguished between negative control dentifrices and proven positive control dentifrices such as those containing Tcs and SnF. Additionally, indirect and direct evidence was combined in a NMA to provide a more precise estimates of treatment effects.^19^, ^75^, ^76^ However, in the NMA of studies with a follow‐up, problems of heterogeneity and potential inconsistency are present which emphasizes that conclusions about ranking should be carefully interpreted.

Interestingly, in the descriptive summary (see Table 2) of the studies with a follow‐up, the results were not in favour of Tcs or SnF, with the only exception in one comparison when the control was SnF‐DF.^46^ In systematic reviews evaluating the efficacy of Tcs or SnF, these active ingredients generally showed better results for plaque and gingival index scores than conventional dentifrices.^10^, ^11^, ^79^, ^80^ In ranking the treatments in studies with a follow‐up according to the NMA (see Appendix S11), the efficacy of Tcs or SnF was in line with the findings of the systematic reviews discussed above.

In addition to the difference of means (DiffM) and 95% confidence intervals, we calculated 95% prediction intervals. The advantage of prediction intervals is they reflect the variation in treatment effects across different settings, including what effect is to be expected in future patients.^15^ The prediction interval of the single‐brushing studies indicated that in a future single‐brushing setting, the difference in means for end plaque scores would likely be between −0.28 and −0.11 (Table 3A) if compared with a negative control as recorded on a five‐point scale according to TQ&H. Compared to a positive control, this will most likely be between −0.33 and −0.03 (Table 3A). Consequently, the probability that in future studies the effect size is less than the threshold 0 is quite certain for both negative and positive controls.^15^

The favourable effect of BS on plaque was not substantiated in studies with a follow‐up. Table 3 shows eight MA that yielded significant results in a follow‐up comparison. However, all comparisons had a 95% prediction interval that included the null, and the seventh comparison examined a group of dentifrices of which three out of five were not available on the market. Nevertheless, based on the prediction intervals, the probability^15^ is that 84% to 96% of the participants in future studies can expect a small effect on bleeding scores.

This SR follows the recommendation to provide the 95% confidence intervals around *I* ^2^, given that *I* ^2^ itself is not precise.^84^ Values of *I* ^2^ ranging from 0% to 100% inform us what proportion of the total variation across studies is beyond chance.^84^ With a small number of included studies, *I* ^2^ has low statistical power and its confidence intervals can be large with upper 95% confidence intervals that cross into the range of large heterogeneity (*I* ^2^ ≥50%).^84^, ^85^ An example appears in Table 3B3, in which a heterogeneity of 14% shows the upper limit of the 95% confidence intervals in the range of large heterogeneity (87%). Without the interval, one might erroneously assume low heterogeneity. Nonetheless, it is not unreasonable to assume that the overall external validity was reasonably. Considering all aspects, the overall judgement of the risk of bias for all included studies was estimated to be moderate. Details are provided in Table 4.

Putt et al^49^ investigated the effect of varying concentrations of BS. The results suggested a possible positive relationship between the concentration of BS and plaque reduction. In the ranking of the NMA results (Online Appendix S11‐C), such a pattern is clearly visible. An almost inverse relationship exists between the percentage of BS in a dentifrice and its abrasiveness.^86^ However, a key difference between BS and common abrasives is the size of the particles.^49^ In the case of BS crystals, the particles are notably larger, softer and potentially less damaging to tooth mineral than the conventional abrasive particles in other dentifrices. This size could play a role in disturbing the adhesion of plaque to the tooth surface, in addition to the force exerted by the toothbrush.^49^ The dissolved bicarbonate ions in BS are thought to bind with calcium ions, disrupting the mutual bond between bacteria and disrupting the attachment of bacteria to the tooth surface. These bicarbonate ions are also thought to charge the tooth surface negatively, which enhances the detachment of bacteria.^50^ Furthermore, BS is an alkali, which boosts the cleansing activity of the surfactant in the dentifrice.^50^ Although these proposed mechanisms are promising, BS is known to be easily soluble and slow‐acting. Therefore, it is unlikely to reside long enough in the mouth to actually inhibit plaque growth.^49^, ^86^, ^87^

A recent systematic review found, with moderate certainty, that the adjunctive use of a standard fluoride dentifrice with toothbrushing did not contribute to the effectiveness of mechanical removal of dental plaque, in single‐brushing experiments.^74^ Given that in single‐use studies, BS showed a positive effect in instant plaque removal, incorporating this ingredient into novel dentifrices seems therefore an interesting approach to improve a product.

Other results indicate that BS in dentifrice is an effective buffering agent through its ability to increase pH to a safe, neutral level.^2^, ^89^ A long‐term in situ crossover study showed that BS did not significantly enhance the ability of fluoride dentifrice to reduce demineralization and increase remineralization of the enamel. Most BS‐based dentifrices contain fluoride, which is compatible with BS.^90^, ^91^ Findings from in vitro studies suggest, however, that adding BS to a dentifrice may interfere with the reactivity of fluoride with enamel, reducing mainly the concentration of CaF2 formed.^92^, ^93^ This indicates that adding BS to dentifrice requires careful formulation.

### 5.1 Limitations

An important limitation of this review was the variability of dentifrice formulations in the included papers. The composition of the studied dentifrices was often not clear. The choice of a control dentifrice with which to compare dentifrices formulated for plaque control is also important and could affect conclusions drawn from clinical trials of such products.^94^ In most studies, great effort was invested in creating neutral packaging. However, the unique properties of BS make blinding relatively hard. BS has the reputable property of an odd taste and texture.^49^ This makes a BS‐DF easily distinguishable, especially to participants who are accustomed to a regular fluoride dentifrice.

Only 6 out of 21 studies provided information about allocation concealment, a critical design feature to minimize bias.

The majority of the studies were published between 1986 and 2011, and in most cases, the manner of reporting did not follow current standards, such as TIDieR 2014.^95^ Also, more recently published studies contain data from over 5 years ago.^53^ This limitation is also reflected in the results of the risk of bias assessment. However, all groups seem to have been treated equally and in most of the studies seem to have been well balanced. Unclear in the included studies were the instructions on brushing duration and brushing frequency, details concerning the toothbrushes and the study procedures.

## 6 CONCLUSION

BS‐DF showed promising results with respect to plaque removal in single‐use studies. However, the finding was partially substantiated in follow‐up studies. Studies that assessed bleeding scores indicated that a small reduction can be expected from BS, relative to a control product.

## 7 CLINICAL RELEVANCE

### 7.1 Scientific rationale for the study

Twice daily toothbrushing with a fluoride dentifrice is a universal recommendation for personal oral care.

### 7.2 Principal findings

With moderate certainty, a dentifrice containing BS is comparable to other commercially available dentifrices for controlling plaque and gingivitis.

### 7.3 Practical implications

In order to remove plaque and improve gingival health, toothbrushing can be combined with a baking soda dentifrice.

## CONFLICT OF INTEREST

The authors declare that they have no conflicts of interest. Van der Weijden, Slot and their research team at ACTA have previously received either external advisor fees, lecturer fees or research grants from toothbrush and dentifrice manufacturers. Those manufacturers included: Colgate, Dentaid, GABA, GSK, Lactona, Oral‐B, Procter & Gamble, Sara Lee, Sunstar and Unilever. Ethical approval was not required.

## AUTHOR CONTRIBUTIONS

All authors gave final approval and agreed to be accountable for all aspects of the work ensuring integrity and accuracy. CV contributed to design, search and selection, analysis and interpretation, and drafted the manuscript. YK and AD involved in search and selection, contributed to analysis and drafted the preliminary manuscript. GAW contributed to conception and design, analysis and interpretation, and critically revised the manuscript. DES contributed to conception and design, search and selection, analysis and interpretation, and critically revised the manuscript.
