# Supplementary material for: The efficacy of baking soda dentifrice in controlling plaque and gingivitis: A systematic review
Source: Int J Dent Hyg. 2019 Mar 6;17(2):99–116. doi: 10.1111/idh.12390 (PMC6850485; doi:10.1111/idh.12390)
Supplement: Supplementary file 1 [file IDH-17-99-s001.docx]

**ppendix S1.**

**Post hoc changes to the protocol.**

- Several subgroup and sensitivity analyses were planned but could not be performed due to limited data.

- As sub-analysis, network meta-analyses were performed post hoc and are appropriately noted. The Cochrane Handbook (6) expresses a preference for direct evidence, highlighting the observational nature of indirect evidence, and encourages the use of indirect evidence as a supplemental analysis (78).

A network meta-analysis (NMA) is a method to build on findings from a conventional meta-analysis (22). When multiple interventions have been used and compared for the same disease and outcomes, NMA offers a set of methods to visualize and interpret the wider picture of the evidence and to understand the relative merits of these multiple interventions (19, 75, 76). NMA enables investigators to compare the effects of multiple health care interventions including treatments that were not previously compared in head-to-head trials. Additionally, combining indirect and direct evidences can sometimes provide more precise estimates of treatment effects to support decision-making (77). NMA has been recommended as the highest level of evidence for treatment guidelines (21, 22). Zooming out toward larger scales of evidence may help us to understand the strengths and limitations of the data guiding the medical care of individual patients (19).

The comparisons between different treatments constitute a network (18). For a valid analysis of the network, consistent information from different pathways is assumed (29).

Important in understanding a NMA is assessing the network geometry, finding out which interventions are included in the network and figuring out which interventions have been compared against each other in randomized trials (18, 20). The geometry of the network may show that not all treatments represented equally in the network (18), that some (head to head) comparisons have been ignored or that some comparators were preferred (19, 20).

One appealing feature of a NMA is the rank ordering of interventions (20). However, an intervention may be ranked highly even though it was assessed in only a few trials and with a few patients, which resulted in a misleadingly strong endorsement for the intervention despite large uncertainty (20). Clinical researchers and physicians should always be able to examine the uncertainty in the ranking of interventions, because the difference between interventions might be small and not clinically relevant (20).

Direct and indirect treatment estimates are calculated in netmeta. This function combines and prints these estimates. A comparison of direct and indirect treatment estimates can serve as a check for consistency of NMA (28). This function provides a back-calculation method to derive indirect estimates from direct pairwise comparisons and network estimates. The direct evidence proportion as described in König et al. (2013) (27) is used to calculate the indirect evidence (24).

Another specific issue in NMA is the importance of the transitivity assumption to validate NMA. The distribution of effect modifiers should be similar (11, 30). This was accomplished by an analysis on ingredients. An exception to this is the group Non-BS, the exact composition of which is not clear.

**ONLINE Appendix S2. Overview of the studies processed for data extraction.**

| **Authors** (**year)**  **Study design, duration** | **# Participants baseline (end),**  **gender,**  **age (mean/range),**  **oral prophylaxis (OP)** | **Groups**  ***Brands*** | **Regimen:**  **use &**  **instructions** | **Conclusions of the original authors** |
| --- | --- | --- | --- | --- |
| **Akwagyiram et al. (2018) (37)**  RCT  Parallel (6m)  Full mouth  12 hours of no oral hygiene procedures | 247(228)  Population Silverstone Research Group, Las Vegas, NV, USA  ♀: 141♦(57.1%)  ♂: 106♦(42.9%♦)  Mean(sd) age:  36.0(13.63)  Age range: 18-79  Smoking status: reported  OP: yes (after baseline) | TB+ BS-67% DF  TB+ NaF-DF  **BS-67% DF:** 67% sodium bicarbonate, sodium fluoride; experimental  RDA: *NR*  Fluoride: 1150 ppm F  **NaF DF**: sodium fluoride; Crest Cavity Protection; Procter & Gamble Co; USA  RDA: *NR*  Fluoride: 1100 ppm F  TB: Oral-B^®^ 35 Soft Standard Toothbrush; Procter & Gamble Co., USA) | Participants had to have at least 20 bleeding points at baseline.  Self-brushing, own technique, twice a day for 1 minute.  Refraining from other forms of oral hygiene except for flossing if this was part of the subject’s usual oral hygiene routine.  Prior to each visit, participants were instructed not to consume anything for at least four hours | Twice-daily brushing with sodium bicarbonate containing dentifrices significantly improved indices of gingival bleeding and plaque compared with a non-BS dentifrice in subjects with moderate gingivitis. |
| **Bosma et al. (2018) (38)**  RCT  Crossover (w.o. 3 days)  Single-brushing exercise  Full mouth (PI results divided in six sites)  24 hours of no oral hygiene procedures | 56(56)  Population Salus Research, Inc., Fort Wayne, IN, USA  ♀: 36 (64.3%)  ♂: 20 (35.7%)  Mean(sd) age:  44.1(11.87)  Age range: 19-66  Smoking: no exclusion criteria  OP: *NR* | TB+ BS-67% DF  TB+ BS-67% DF  TB+ BS-62% DF  TB+ NaF-DF  **A) BS-67% DF:** **herbal** mixture, 67% sodium bicarbonate, sodium fluoride; experimental  RDA: *NR*  Fluoride: 923 ppm F  **B) BS-67% DF:** 67% sodium bicarbonate, sodium fluoride; experimental  RDA: *NR*  Fluoride: 923 ppm F  **C) BS-62% DF:** **herbal** mixture, 67% sodium bicarbonate, sodium fluoride; experimental  RDA: *NR*  Fluoride: 923 ppm F  **D) NaF DF**: sodium fluoride; experimental  RDA: *NR*  Fluoride: 923 ppm F  TB: Oral-B^®^ 40 Soft Compact Toothbrush; Procter & Gamble Co., Cincinnati, OH, USA) | Participants had to be a whole mouth PI ≥ 2.00 based on the TQ&H.  Refraining from other forms of oral hygiene.  Single-brushing exercise under supervision with 1.5 g of the assigned dentifrice for 60 seconds at the study site.  Prior to each visit, participants were instructed not to consume anything for at least four hours. | Plaque removal was significantly greater with sodium bicarbonate containing dentifrices compared with a non-BS dentifrice after a single, timed brushing. There was no effect of herbal tinctures. |
| **Jose et al. (2018) (54)**  RCT  Parallel (6m)  Full mouth  24 hours of no oral hygiene procedures | 246 (216◊)  Population Salus Research, Inc., Fort Wayne, IN, USA  ♀: 152 (61.8%)  ♂: 94 (38.2%)  Mean(sd) age:  38.58(13.5)  Age range: 18-?  Smoking: no exclusion criteria  OP: optional | TB+ BS-67% DF  TB+ NaF-DF  TB: Oral-B^®^ 35 Soft Standard Toothbrush; Procter & Gamble Co., Cincinnati, OH, USA)  **BS-67% DF:** 67% sodium bicarbonate, sodium fluoride; experimental; GSK Consumer Healthcare, Weybridge, Surrey, UK  RDA: *NR*  Fluoride: 1,150 ppm F  **NaF-DF:** sodium fluoride; Colgate^®^ Triple Action; Colgate-Palmolive Co, New York, NY, USA)  RDA: *NR*  Fluoride: 1,150 ppm F | Participants had to be a whole mouth BI ≥ 20 bleeding sites based on the modified BI (68).  Self-brushing, own technique, twice a day for 1 minute. | Gingival bleeding, gingivitis, and plaque indices were significantly improved at 24 weeks with twice-daily brushing with a 67% sodium bicarbonate dentifrice in participants with moderate gingivitis. |
| **Hosadurga et al. (2017) (36)**  RCT  Parallel (1m)  Full mouth  Hours of no oral hygiene procedure: *NR* | 50 (45◊)  Patients Yenepoya Dental College, Mangalore, India  ♀: 30◊ (66.7%◊)  ♂: 15◊ (33.3%◊)  Mean(sd) age:  24.1◊(8.3◊)  Age range: 18-?  Smoking: exclusion criteria  OP: no | TB+ BS-DF  TB+ MFP-DF  TB: *NR*  **BS-DF:** herbal mixture, sodium bicarbonate, sodium fluoride, Parodontax® (GlaxoSmithKline, Middlesex, United Kingdom)  RDA: *NR*  Fluoride: 1,400 ppm F  **MFP-DF:** herbal mixture, calcium carbonate, Colgate® Herbal (Colgate- Palmolive India Limited, Mumbai, India)  RDA: *NR*  Fluoride: *NR* | Participants had to be a whole mouth PI ≥ 1.5 based on the TQ&H and a GI ≥ 1.0 based on the Löe-Silness Gingival Index.  Self-brushing, own technique, twice a day for a period of 5-10 minutes. | There was no significant difference between the groups in GI and PI levels. |
| **Mason et al. (2017) (53)**  RCT(2X)  Crossover (w.o. 3 days)  Single-center  Full mouth  Single-brushing exercise  24 hours of no oral hygiene procedures | For all sub-studies:  Population: *NR*  Location: Research Center, Cheshire, UK  Smoking status: *NR*  OP: no | For all sub-studies:  TB: (Oral-B® 35 Indicator, Compact, Soft; Procter & Gamble Co., Cincinnati, OH, USA)? | Participants had to be a whole mouth PI ≥ 2.00 based on the TQ&H.  Refraining from other forms of oral hygiene.  Single-brushing exercise under supervision with 1.5 g of the assigned dentifrice for 60 seconds at the study site.  Prior to each visit, participants were instructed not to consume anything for at least four hours. | The commercially available dentifrices containing 67% or 45% sodium bicarbonate  exerted a significantly greater effect on plaque removal than commercially available dentifrices without sodium bicarbonate. |
|  | **Sub-study 1:**  60 (60)    ♀: 41◊(68,3%)  ♂: 19◊(31.7%◊)  Mean(sd) age: 40.4(10.93)  Age range: 18-? | **Sub-study 1:**  TB+ BS-67% DF  TB+ BS-45% DF  TB+ NaF-DF  **A) BS-67% DF****:** 67% sodium bicarbonate, sodium fluoride with herbs; Parodontax ®-F *  RDA: 68  Fluoride: 1,425 ppm F  **B) BS-45% DF:** 45% sodium bicarbonate, sodium fluoride with herbs and silica; Parodontax® Gentle Whitening *  RDA: 107  Fluoride: 1,425 ppm F  **C)** **NaF-DF:** sodium fluoride; Crest® Decay prevention **  RDA: 129  Fluoride: 1,425 ppm F  * GSK Consumer Healthcare, Weybridge, Surrey, UK  ** Procter & Gamble Co., Cincinnati, OH, USA |  |  |
|  | **Sub-study 2:**  55 (54◊)    ♀: 41◊(74.5%)  ♂: 14◊(25.5%◊)  Mean(sd) age: 37.3(11.41)  Age range: 18-? | **Sub-study 2:**  TB+ BS-67% DF  TB+ SnF-DF  **BS-67% DF:** 67% sodium bicarbonate, sodium fluoride with herbs; Parodontax ®-F *  RDA: 68  Fluoride: 1,425 ppm F  **SnF-DF:** stannous fluoride, amine fluoride with silica; Meridol® ♦  RDA: 55  Fluoride: 1,400 ppm F |  |  |
| **Lomax et al. (2016) (52)**  RCT  Parallel (6w)  Single-center  Full mouth  24 hours of no oral hygiene procedures | 148 (135◊)  Non-dental: ?  New Delhi (India)?  ♀: 84◊ (56.8%◊)  ♂: 64◊ (43.2%◊)  Mean(sd) age:  28.2◊(9.14◊)  Age range: 18-?  Smoking status: reported  OP: yes (after baseline) | TB+ BS DF  TB+ NaF-DF  TB: Medium toothbrush  (provided)  **BS-DF**: 67% sodium bicarbonate, sodium fluoride; Parodontax® Daily Toothpaste (GlaxoSmithKline Asia Pvt. Ltd, Punjab, India)  RDA:  *NR*  Fluoride: 923 ppm F    **NaF-DF**: Experimental non-sodium bicarbonate, silica sodium fluoride; DF, not commercially available.  RDA:  *NR*  Fluoride: 923 ppm F | Participants with mild-to-moderate gingivitis, a positive response to bleeding on brushing and ≥ 20 bleeding sites.  Self-brushing,  own technique, twice a day.    Refraining from other forms of oral hygiene  11 p.m. on the evening prior to each visit, participants were instructed not to consume anything. | A 67% BS dentifrice provided statistically significant improvements in gingival health and bleeding after 6 weeks of use compared to the NaF dentifrice. |
| **Triratana et al. (2015) (51)**  RCT  Parallel (6m)  Single-center  Full mouth  Hours of no oral hygiene procedure: *NR* | 135 (135◊)  Non-dental: ?  Bangkok (Thailand)  ♀: 69◊ (51.1%◊)  ♂: 66◊ (48.9%◊)  Mean(sd) age:  38.5◊ (9.15◊)  Age range: 18-70  Smoking status: *NR*  OP: *NR* | TB+ BS-DF  TB+ MFP+NaF-DF  TB+ Tcs-DF  TB: Soft-bristle toothbrush (provided)    **BS-DF:** herbal mixture, bicarbonate, sodium fluoride; Parodontax®  RDA: *NR*  Fluoride: 1,400 ppm F    **A) MFP+NaF-DF**: sodium fluoride and monofluorophosphate; Colgate Cavity Protection®  RDA: *NR*  Fluoride: 450 ppm F (sodium fluoride) and 1,000ppm F (sodium monofluorophosphate).    **B) Tcs-DF**: 0.3% triclosan, 2.0% copolymer, silica sodium fluoride; Colgate Total ®;  RDA: *NR*  Fluoride: 1,450 ppm F- | Participants had to be a whole mouth PI ≥ 1.5 based on the TQ&H and a GI ≥ 1.0 based on the Löe-Silness Gingival Index.  Self-brushing, own technique.    Refraining from other forms of oral hygiene  Brush one minute twice-daily (morning and evening) with the assigned TB and dentifrice. | The triclosan/copolymer/fluoride dentifrice was superior to the BS dentifrice.  The BS dentifrice was equivalent to the fluoride dentifrice in its effect on plaque and gingivitis over 6 months of product use. |
| **Al-Kholani (2011) (35)**  RCT  Parallel (6w)  single-center  Full mouth?  Hours of no oral hygiene procedures: *NR* | 48 (*NR*)  Population: patients with chronic marginal gingivitis  from a private clinic in Sana’a, Yemen  ♀: *NR*  ♂: *NR*  Mean age: *NR*  Age range: *20-40*  Smoking status: *NR*  OP: *yes* (after baseline) | TB+ BS-DF  TB+ NaF-DF  TB+ Non-BS Herbal DF  TB: *NR*  **BS-DF:** sodium bicarbonate, sodium fluoride, herbal ingredients (chamomile, echinacea, sage rhatany, myrrh, peppermint oil); Parodontax®; (GlaxoSmithKline, Middlesex, United Kingdom)  RDA: *NR*  Fluoride: 1400ppm F-  **A) Non-BS Herbal-DF:** herbal ingredients (chamomile, calendula, sage); Silca® Herbal◊ Toothpaste  **B) NaF-DF:** 0,32% sodium fluoride, hydrated silica, other ingredients (sorbitol, water, SLS, Peg-32, flavor, cellulose gum, sodium saccharin, trisodium phosphate, vitamin-E-acetate)  RDA: *NR*  Fluoride: 1450 F-◊ | Participants with chronic marginal gingivitis.  Self-brushing, given oral hygiene instruction.  Toothbrushing for two minutes, three times/day  Refraining from other forms of oral hygiene: *NR* | The BS dentifrice was more effective for the reduction of dental plaque than with the (non-BS) herbal dentifrice; however, differences were non-significant  Signs of gingival inflammation reduced significantly in both herbal test groups compared to the conventional group. |
| **Ghassemi et al. (2008)** (50)  RCT  Parallel (4w) &  Single-brushing exercise:   1. Baseline single-brushing 2. End single-brushing   Single-center  Full mouth    24 hours of no oral hygiene procedures | 218 (207)  Non-dental: ?  University Park Research Center, Fort Wayne, IN, (USA)?    Sex of participants who finished the study:  ♀: 144(66.1%◊)  ♂: 63(28.9%◊)  Mean age: 39  Age range: 18-71    Smoking status: *NR*  OP: *NR* | TB+ BS-DF  TB+ Tcs-DF  TB: (Oral-B 35 Compact Soft)    **BS-DF:** BS; Arm & Hammer® Advance White® Brilliant Sparkle; (Church & Dwight Co., Inc., Princeton, NJ, USA)  RDA: *NR*  Fluoride: ? ppm F-    **Tcs-DF:** 0.3% triclosan/ 2.0% copolymer; Colgate® Total® Clean Mint Dentifrice; (Colgate-Palmolive Company, New York, NY, USA)  RDA: *NR*  Fluoride: ? ppm F- | Participants had to be a whole mouth PI ≥ 2 based on the TQ&H.  Self-brushing at home, own technique; 60 seconds twice-daily.    Refraining from other forms of oral hygiene.  At baseline visit and 4w visit (supervised): Single-brushing with 1.5 g of the assigned dentifrice for 60 seconds at the study site. | The BS dentifrice was significantly more effective in reducing and controlling plaque accumulation compared to a clinically proven antimicrobial (triclosan/copolymer) dentifrice. |
| **Putt et al. (2008) (49)**  RCT (5X)  Crossover (w.o. 3 to 5 days)  Single-brushing exercise  Multi-center  Full mouth  Participants refrained from oral hygiene for 24 hours, and did not eat, drink, or smoke the morning of their examination visit. | For all sub-studies:  Population: *NR*  Location: *NR*  Smoking status: *NR*  OP: *NR* | For all sub-studies:  TB: Oral-B® 35 Compact Toothbrush; ***  RDA: *NR*  Fluoride: *NR* ppm F- | Participants had to be a whole mouth PI ≥ 1.5 based on the TQ&H and had no periodontitis affecting more than two teeth.  Self-brushing, own technique.  Each visit involved a baseline (pre-brushing) plaque assessment, a one-minute supervised brushing with the assigned test dentifrice, and a post brushing plaque assessment.  Refraining from other oral hygiene products: *NR*.  During each w.o. (3 to 5 days): follow normal oral hygiene procedures using BS-free fluoride DF and toothbrush provided. | The sodium bicarbonate dentifrices enhanced plaque removal effectiveness of toothbrushing to a significantly greater extent than the non- BS dentifrice products. |
|  | **Sub-study 1:**  71 (65)    ♀: 50(76.9%◊)  ♂: 15(23.1%◊)  Mean(sd) age: 42(13)  Age range: 18-68 | **Sub-study 1:**  TB+ BS-65% DF  TB+ BS-20% DF  TB+ Tcs-DF  **A) BS-65%:** BS 65%; Arm & Hammer® Dental Care®; *  **B) BS-20%:** BS 20%;  Arm & Hammer® Advance White™ Brilliant Sparkle; *    **C) Tcs-DF:** 0.3% triclosan, copolymer; Colgate® Total® Clean Mint; ** |  |  |
|  | **Sub-study 2:**  68 (66)  ♀: 49(74.2%◊)  ♂: 17(25.8%◊)  Mean(sd): 40(13)  Age range: 22-81 | **Sub-study 2:**  TB+ BS-48% DF  TB+ BS-20% DF  TB+ Tcs-DF  TB+ SnF-DF   1. **BS-48% DF:** BS 48%;   Arm & Hammer® Advance White™ BS and Peroxide; *   1. **BS-20% DF:** BS 20%;   Arm & Hammer Advance White Brilliant Sparkle; *  **C) Tcs-DF:** 0.3% triclosan, copolymer; Colgate® Total® Clean Mint; **  **D) SnF-DF:** stannous fluoride and sodium hexametaphosphate; Crest® Pro-Health™; *** |  |  |
|  | **Sub-study 3:**  69 (64)  ♀: 46(71.9%◊)  ♂: 18(28.1%◊)   Mean(sd) age: 41(11)  Age range: 22-68 | **Sub-study 3:**  TB+ BS-48% DF  TB+ BS-27% DF  TB+ Tcs-DF  TB+ NaF-DF  **A) BS-48% DF:** BS 48%; Arm & Hammer® Advance White™ BS and Peroxide; *  **B) BS-27% DF:** BS 27%; Arm & Hammer® Advance White™ Liquid Gel; *  **C) Tcs-DF:** Colgate® Total® Clean Mint; 0.3% triclosan, copolymer; **  **D) NaF-DF:** sodium fluoride, silica; Crest® Regular Cavity Protection; *** |  |  |
|  | **Sub-study 4:**  36 (36)  ♀: 27(75%◊)  ♂: 9(25%◊)  Mean(sd): 38(11)  Age range: 19-64 | **Sub-study 4:**  TB+ BS-65% DF  TB+ NaF-DF  **BS-65% DF:** BS 65%;  Arm & Hammer® Dental Care®; *  **NaF-DF:** sodium fluoride, silica Aim® Cavity Protection Gel; * |  |  |
|  | **Sub-study 5:**  45 (41)  ♀: 33(80.5%◊)  ♂: 8(19.5%◊)  Mean(sd): 42(12)  Age range: 20-64 | **Sub-study 5:**  TB+ BS-20% DF  TB+ Tcs-DF  **BS-20% DF:** BS 20%;  Arm & Hammer Advance White Brilliant Sparkle; *  **Tcs-DF:** 0.3% triclosan, copolymer; Colgate® Total® Clean Mint; **  *Church & Dwight Co., Inc., Princeton, NJ, USA  **Colgate-Palmolive Company, New York, NY, USA  ***Procter & Gamble, Cincinnati, OH, USA |  |  |
| **Ozaki et al. (2006) (48)**  RCT  Parallel (4w)  Single-center  Full mouth (GI and PI results divided in lingual+ buccal and mesial+ distal)  Hours of no oral hygiene procedures: *NR* | 48 (42)  Dental Clinic  São Caetano (Brazil)  ♀: 28(58.3%◊)  ♂: 20(41.7%◊)  Mean(sd) age: 33.19(13.57)  Age range: 18-69  Smoking status: *NR*  OP: *NR* | TB+ BS-DF  TB+ Tcs-DF  TB: *NR*  **BS-DF:** sodium bicarbonate, sodium fluoride, herbal ingredients (chamomile, echinacea, sage, rhatany, myrrh and peppermint oil); Parodontax® (GlaxoSmithKline, Middlesex, United Kingdom)  RDA: *NR*  Fluoride: 1,400 ppm F-  **Tcs-DF:** containing 0.3% triclosan, 2% copolymer and 0.243% sodium fluoride; Colgate Total ® (Colgate-Palmolive Company, New York, United States of America); Colour and taste modified similar to Parodontax ®  RDA: *NR*  Fluoride: 1,400 ppm F- ◊ | Participants had to be a whole mouth PI ≥ 1.5 based on the TQ&H, a GI ≥ 1.0 based on the Löe-Silness Gingival Index and presence of established gingivitis.  Self-brushing, own technique  The participants were instructed to brush with the assigned dentifrice three times a day. Rinsing with water after toothbrushing was not allowed, as well as using any other dentifrice or mouthrinse during the experimental period.  All participants used dental floss during the study. | Both dentifrices were able to reduce plaque and gingivitis, although there was no significant difference between the two observed groups. |
| **Mankodi et al.** **(1998)** (47)  RCT  Crossover (w.o. 1w)  Single-brushing exercise  partial mouth  48 hours no oral hygiene procedures | 34 (*NR*)  Population: NR  Location: *NR*  ♀: *NR*  ♂: *NR*  Mean age: *NR*  Age range: 18-65    Smoking status: *NR*  OP: *NR* | TB+ BS-DF  TB+ NaF-DF  TB+ MFP+NaF-DF  TB: NR  **BS-DF:** 65% sodium bicarbonate; Arm & Hammer Dental Care® (Church & Dwight Co., Inc., Princeton, NJ)  RDA: 30-40  Fluoride: *NR*  **NaF-DF:** hydrated silica, sodium fluoride ◊; Crest® Regular DF (Procter & Gamble, Cincinnati, OH)  RDA: 100-110  Fluoride: *NR* ppm F-  **MFP+NaF-DF:** dicalcium phosphate, (monofluorophosphate & sodium fluoride?); Colgate® Regular Toothpaste (Colgate-Palmolive Co., New York, NY)  RDA: 70-80  Fluoride: *NR* ppm F- | Self-brushing, own technique under supervision.    Refraining from other forms of oral hygiene | BS is an effective dentifrice abrasive system for removing accumulated plaque and maintaining good oral health. superior to both other dentifrices tested. |
| **Beiswanger et al. (1997) (46)**  CCT  Parallel (6m)  Full mouth  Single-center  Hours of no oral hygiene procedures: *NR* | 570 (554)  Indianapolis metropolitan area (Indiana, USA)  ♀: 413◊(72.5%◊)  ♂: 157◊(27.5%◊)  Mean(sd) age: 36.3◊(10.5◊)  Age range: *NR*  Smoking status: *NR*  OP: yes (after baseline) | TB+ BS-DF  TB+ NaF-DF  TB+ SnF-DF  TB: *NR*  **BS-DF:** BS, peroxide, 0.243% sodium fluoride; Mentadent® Fluoride dentifrice; (Chesebrough-Ponds, Greenwich, CT).  RDA: *NR*  Fluoride: 1,100 ppm F-◊  **A) NaF-DF:** 0.243% sodium fluoride, silica; Crest® Regular  RDA: NR  Fluoride: 1,100 ppm F-◊  **B) SnF-DF:** 0.454% stabilized stannous fluoride, silica; Crest® Plus Gum Care; (Procter & Gamble, Cincinnati, OH, USA◊)  RDA: *NR*  Fluoride: 1,000◊ ppm F- | Participants did have at least 5 gingival bleeding sites based on the Löe-Silness Index.  New toothbrush provided at baseline and three-month examination.  Self-brushing at home, own technique  Brushing twice-daily  Refraining from other dentifrices, mouthrinses or oral irrigation products. | The sodium bicarbonate and NaF/peroxide dentifrice did not provide reductions in gingivitis, plaque or gingival bleeding as compared with the conventional NaF dentifrices. |
| **Mullally et al. (1995) (5)**  RCT  Parallel (6w)  Single-center  Partial mouth (index teeth)  Hours of no oral hygiene procedures: *NR* | 70 (70)  Northern-Ireland?  Belfast (Northern-Ireland)    ♀: 36(51.4%◊)  ♂: 34(48.6%◊)  Mean age: *NR*  Age range: 18-65  Smoking status: *NR*  OP: yes (after baseline and at the end of the study) | TB+ BS-DF  TB+ MFP+NaF-DF    TB: Oral B 35 (Oral B laboratories, Aylesbury, Bucks, England)    **BS-DF:** sodium bicarbonate, herbal ingredients (camomile, echinacea, sage, myrrh, rhatany, peppermint oil); Parodontax® (Madaus, Cologne, Germany)  RDA: *NR*  Fluoride: *NR* ppm F-  **MFP+NaF-DF:** monofluorophosphate◊, sodium fluoride◊; Colgate (Colgate-Palmolive, London, England)  RDA: *NR*  Fluoride: *NR* ppm F- | Participants had to be a whole mouth PI > 2 based on the TQ&H.  Self-brushing,  own technique, twice a day.  Refraining from other oral hygiene products.  Participants were asked to keep a diary to record compliance with the brushing instructions | The herbal based (sodium bicarbonate) dentifrice is as effective as the conventionally formulated dentifrice in the control of plaque and gingivitis, however, it has no significant clinical advantage over a traditionally formulated dentifrice. |
| **Saxer et al. (1995)** (45)  RCT  Parallel (2m)  Single Centre  Half mouth,  contralateral  Hours of no oral hygiene procedures: *NR* | 60 (46◊)  Factory workers  Köln (Germany)?    ♀: *NR*  ♂: *NR*  Mean age: *NR*  Age range: *NR*  Smoking status: *NR*  OP: *NR* | TB+ BS-DF  TB+ Non-BS Herbal-DF  TB: Oral-B35  **BS-DF:** sodium bicarbonate, herbal ingredients; Parodontax®; (Madaus AG, Cologne, Germany)  RDA: *NR*  Fluoride: *NR* ppm F-  **Non-BS Herbal:** calcium hydrogen phosphate, herbal ingredients, non-marketed new DF  RDA: *NR*  Fluoride: *NR* ppm F- | Participants with clinical signs of gingivitis and no pockets exceeding 5 mm.  Self-brushing own technique    Brushing twice a day.    Refraining from mouthwashes or chewing gum | The amount of plaque did not appear to be influenced by the dentifrices used.    The gingivitis and bleeding indices decreased significantly by 40% in both groups compared to the baseline examination. |
| **Saxer et al. (1994) (44)**  CCT  Parallel (1m)  Full mouth  Single-center  Same examiner  Examiner blinding  Product blinding  Hours of no oral hygiene procedures: *NR* | 22 (22)  Zürich, Switzerland?  BS-P:  ♀: *NR*  ♂: *NR*  Mean age: 30.3  Age range: *NR*    F-DF:  ♀: *NR*  ♂: *NR*  Mean age: 31.0  Age range: *NR*    Smoking status: *NR*  OP: *NR* | TB+ BS-DF  TB+ Non-BS F-DF  TB: Parodontax® toothbrush  **BS-DF:** Parodontax® ◊  RDA: *NR*  Fluoride: *NR* ppm F-  **Non-BS F-DF:** a marketed fluoride DF; brand?  RDA: *NR*  Fluoride: *NR* ppm F- | Participants with clinical signs of gingivitis and no pockets exceeding 5 mm.  Self-brushing at home, own technique    Refraining from other forms of oral hygiene: *NR*    Brushing twice a day. | There was a significant decrease found for the sodium bicarbonate dentifrice in BOP compared to the control group and baseline    There was a significant decrease found for the sodium bicarbonate dentifrice in bleeding sites compared to control group and baseline |
| **Taller (1993) (43)**  RCT  Parallel (5w)  Full mouth (examined 6 teeth)  Same single examiner  Examiner blinding  Product blinding: *NR*  Hours of no oral hygiene procedures: *NR* | 42 (40◊)  Population: *NR*  location: *NR*  ♀: 17(42.5%◊)  ♂: 23(57.5%◊)  Mean age: 46  Age range: 21-71  Smoking status: *NR*  OP: *NR* | TB+ BS-DF  TB+ Non-BS DF  TB: toothbrush + floss provided by the examiner.  **BS-DF:** sodium bicarbonate, sodium◊ fluoride; Mentadent®; Chesebrough-Pond’s Greenwich Ct.  RDA: *NR*  Fluoride: *NR* ppm F-  **Non-BS DF:** participant’s own dentifrice (without BS and CHX)  RDA: *NR*  Fluoride: *NR* ppm F- | Participants had to be at least 20 Bleeding points based on the Ainamo & Bay Index.  Self-brushing, oral hygiene instructions given preliminary.  Not refrained from floss.  Instructed to brush and floss three times a day. | In this study, the sodium bicarbonate dentifrice did not show a significant drop in bleeding points compared to the baseline. Additionally, there was no difference compared to the control groups. |
| **Yankell et al. (1993) (42)**  RCT  Parallel (6m)  Single-center  Full and partial mouth  10-12 hours of no oral hygiene procedures | 128 (123◊)  Population German, Köln (Germany)?  ♀: NR  ♂: *NR*  Mean age: NR  Age range: 18-65 (as inclusion)  Smoking status: *NR*  OP: *NR* | TB+ BS-DF  TB+ Non-BS Herbal-DF  TB: Own toothbrush used  **BS-DF:** sodium bicarbonate, herbal ingredients; Parodontax® (Madaus AG, Cologne, Germany)  RDA:  *NR*  Fluoride: *NR*  **Non-BS Herbal-DF:** calcium carbonate, herbal ingredients◊; non-marketed modified (similar consistency, colour, taste as Parodontax®); (Madaus AG, Cologne, Germany)  RDA: *NR*  Fluoride: *NR* ppm F- | Self-brushing for 6 months,  own technique, twice a day with the assigned dentifrice.  Refrained to use any commercial mouthrinse or other oral hygiene solutions with the exception of floss. | The sodium bicarbonate dentifrice was found to be significantly more effective in reducing plaque gingivitis and bleeding on probing than the placebo. |
| **Emling and Yankell (1988) (41)**  RCT  Parallel  Single-brushing exercise  Partial mouth  16-18 hours of no oral hygiene procedures | 12 (*NR*)  Population: ?, Philadelphia, Pennsylvania USA?  ♀: *NR*  ♂: *NR*  Mean age: *NR*  Age range: *NR*  Smoking status: *NR*  OP: *NR* | TB+ BS-DF  TB+ NaF-DF  TB: *NR*  **BS+DF:** Parodontax®  RDA: *NR*  Fluoride: *NR* ppm F-  **NaF-DF:** sodium fluoride◊; Crest®  RDA: *NR*  Fluoride: *NR* ppm F- | Participants with gingivitis (29%◊) or periodontitis (71%◊).  Self-brushing, own technique  Refraining from other oral hygiene products: *NR* | There was no significant difference found between brushing with the sodium bicarbonate dentifrice and sodium fluoride dentifrice in plaque scores. |
| **Yankell and Emling (1988) (40)**  RCT  Parallel (2m)  Single-center  Partial mouth  10-12 hours of no oral hygiene procedures | ±60◊ (*NR*)  Northern-American  University employees and members of the public at large.  University of Pennsylvania School of Dental Medicine (USA)?    ♀: *NR*  ♂: *NR*  Mean age: *NR*  Age range: *NR*  Smoking status: *NR*  OP: *NR* | TB+ BS-DF  TB+ Non-BS DF  TB+ NaF-DF  TB: Own toothbrush used  **BS-P:** Parodontax®  RDA:  *NR*  Fluoride: *NR* ppm F-  **A) Non-BS DF:** modified dentifrice with laboratory abrasivity indices similar to Parodontax® with no known active ingredients.  RDA: *NR*  Fluoride: *NR* ppm F*-*  **B) Na-DF:** sodium fluoride◊; Crest Tartar Control®  RDA: *NR*  Fluoride: *NR* ppm F- | Self-brushing, own technique, twice a day.  Refraining from other oral hygiene products not reported. | GI: Statistical significant difference favoring the BS dentifrice.  BOP: the BS dentifrice was significantly more effective than both placebo and sodium fluoride dentifrice.  PI: No significant statistical difference was found for the sodium bicarbonate dentifrice, placebo or sodium fluoride dentifrice. |
| **Winer et al. (1986) (39)**  RCT  Parallel (1m)  Single-center  Full mouth  Hours of no oral hygiene procedures: *NR* | 50 (39◊)  Essex county jail, Salem, MA  ♀: 0◊  ♂: 50  Mean age: *NR*  Age range:  18-?  Median age: 24  Smoking status: *NR*  OP: *NR* | TB+ BS-DF  TB+ Non-BS DF  TB: Butler no. 411  **BS-DF:** silica-based, essential oils, sodium bicarbonate, sodium chloride, zinc chloride, and sodium fluoride; Kolynos Periodontal Dental Cream®  RDA: *NR*  Fluoride: *NR* ppm F-  **Non-BS DF:**  hydrated calcium phosphate-base, essential oils.  RDA: *NR*  Fluoride: *NR* ppm F- | Participants with varying degrees of gingival inflammation and plaque accumulation.  Self-brushing, own technique  Brush teeth in usual manner twice a day.  Refrained from all other oral hygiene products.  On average three quarter of a 6-oz. tube was used during the test period. | For both the BS and non-BS dentifrice groups the decrease in the PI was significant. However, there was no significant difference between the two dentifrices. |

◊: calculated by the authors of this review based on the data presented in the selected paper

?: unclear

BOP: Bleeding on Probing

CHX: Chlorhexidine

d: day

DF: Dentifrice

F: Fluoride

GI: Gingival Index

m: month

MFP: Monofluorophosphate

NaF: Sodium fluoride

NR: not reported

OP: Oral Prophylaxis; at the initial appointment, all teeth were thoroughly scaled and polished.

PI: Plaque Index

RDA: Relative (or Radioactive) Dentine Abrasivity (or Abrasion)

SnF: Stannous fluoride

TB: Toothbrush

Tcs: Triclosan

w: week

w.o.: washout period (crossover design)

**Appendix S3: Methodological quality and potential risk of bias scores of the individual included studies.**

|  | | **Akwagyiram et al. (2018) (37)** | **Bosma et al. (2018) (38)** | **Jose et al. (2018) (54)** | **Hosadurga et al. (2017) (36)** | **Mason et al. (2017) (53)** | **Lomax et al. (2016) (52)** | **Triratana et al. (2015) (51)** | **Al-Kholani (2011) (35)** | **Ghassemi et al. (2008) (49, 50)** | **Putt et al. (2008) (49)** | **Ozaki et al. (2006) (48)** | **Mankodi et al. (1998) (47)** | **Beiswanger et al. (1997) (46)** | **Mullally et al. (1995) (5)** | **Saxer et al. (1995) (45)** | **Saxer et al. (1994) (44)** | **Taller (1993) (43)** | **Yankell et al. (1993) (42)** | **Emling and Yankell (1988) (41)** | **Yankell and Emling (1988) (40)** | **Winer et al. (1986) (39)** |
| --- | --- | --- | --- | --- | --- | --- | --- | --- | --- | --- | --- | --- | --- | --- | --- | --- | --- | --- | --- | --- | --- | --- |
| **Internal validity** | **Study design** | **parallel** | **cross-over** | **parallel** | **parallel** | **cross-over** | **parallel** | **parallel** | **parallel** | **parallel** | **cross-over** | **parallel** | **cross-over** | **parallel** | **parallel** | **parallel** | **parallel** | **parallel** | **parallel** | **parallel** | **parallel** | **parallel** |
|  | **Random allocation*** | **+** | **+** | **+** | **+** | **+** | **+** | **+** | **+** | **+** | **+** | **+** | **+** | **-** | **+** | **+** | **-** | **+** | **+** | **+** | **+** | **+** |
|  | **Allocation concealment** | **+** | **+** | **+** | **+** | **+** | **NR** | **NR** | **NR** | **NR** | **NR** | **+** | **NR** | **NA** | **NR** | **NR** | **NA** | **NR** | **NR** | **NR** | **NR** | **-** |
|  | **Blinded to product*** | **+** | **+** | **+** | **+** | **+** | **?** | **+** | **NR** | **NR** | **?** | **+** | **+** | **+** | **+** | **+** | **+** | **-** | **+** | **NR** | **+** | **?** |
|  | **Blinded to examiner*** | **+** | **+** | **+** | **+** | **+** | **+** | **+** | **NR** | **+** | **?** | **+** | **+** | **+** | **+** | **+** | **+** | **+** | **+** | **+** | **+** | **NR** |
|  | **Blinding during statistical analysis** | **+** | **+** | **NR** | **NR** | **+** | **+** | **NR** | **NR** | **NR** | **NR** | **NR** | **NR** | **NR** | **NR** | **NR** | **NR** | **NR** | **NR** | **NR** | **NR** | **NR** |
|  | **Balanced experimental groups*** | **+** | **+** | **+** | **+** | **+** | **+** | **+** | **+** | **+** | **+** | **+** | **+** | **-** | **+** | **+** | **+** | **+** | **?** | **?** | **?** | **+** |
|  | **Reported loss to follow-up*** | **+** | **+** | **+** | **+** | **+** | **+** | **+** | **-** | **+** | **+** | **+** | **-** | **+** | **+** | **+** | **+** | **+** | **+** | **-** | **-** | **+** |
|  | **# (%) of drop-outs** | **19◊(7.7%◊)** | **0(0%)** | **30**◊**(12.2%◊)** | **5 (10 %◊)** | **1◊(0.8%◊)** | **13◊(8.8%◊)** | **0(0%)** | **NR** | **11◊(5.0%◊)** | **17◊(5.9%◊)** | **6◊(12.5%◊)** | **NR** | **16◊(2.8%◊)** | **0(0%)** | **7◊(11.7%◊)** | **0(0%)** | **2◊(4.8%◊)** | **NR** | **NR** | **NR** | **11◊(22%◊)** |
|  | **Treatment identical, except for intervention*** | **+** | **+** | **+** | **+** | **+** | **+** | **+** | **+** | **+** | **+** | **+** | **+** | **+** | **+** | **+** | **+** | **+** | **+** | **+** | **+** | **+** |
| **External validity** | **Representative population group** | **+** | **+** | **+** | **+** | **+** | **+** | **+** | **+** | **+** | **NR** | **+** | **+** | **+** | **+** | **+** | **NR** | **NR** | **+** | **NR** | **+** | **-** |
|  | **Eligibility criteria defined*** | **+** | **+** | **+** | **+** | **+** | **+** | **+** | **+** | **+** | **+** | **+** | **+** | **+** | **+** | **+** | **+** | **+** | **+** | **-** | **-** | **+** |
|  | **Sample size calculation and power** | **+** | **+** | **+** | **+** | **+** | **+** | **+** | **NR** | **NR** | **NR** | **NR** | **NR** | **NR** | **NR** | **NR** | **NR** | **NR** | **NR** | **NR** | **NR** | **NR** |
|  | **Point estimates presented for the primary outcome** | **+** | **+** | **+** | **+** | **+** | **+** | **+** | **+** | **+** | **+** | **+** | **+** | **+** | **+** | **+** | **+** | **+** | **+** | **+** | **+** | **+** |
|  | **Measures of variability presented for the primary outcome** | **+** | **+** | **+** | **+** | **+** | **+** | **+** | **+** | **+** | **+** | **+** | **-** | **+** | **+** | **+** | **+** | **+** | **+** | **+** | **+** | **+** |
|  | **Unit of analysis** | **participant** | | | | | | | | | | | | | | | | | | | | |
|  | **Included a per protocol analysis** | **+** | **+** | **+** | **+** | **+** | **+** | **+** | **NR** | **+** | **+** | **+** | **+** | **+** | **NA** | **?** | **?** | **+** | **+** | **+** | **+** | **+** |
|  | **Included an intention-to-treat analysis** | **+** | **+** | **+** | **-** | **+** | **?** | **-** | **NR** | **-** | **-** | **-** | **-** | **-** | **NA** | **?** | **?** | **-** | **-** | **-** | **-** | **-** |
| **Clinical aspects** | **Validated measurement** | **+** | **+** | **+** | **+** | **+** | **+** | **+** | **+** | **+** | **+** | **+** | **+** | **+** | **+** | **+** | **+** | **+** | **+** | **+** | **+** | **+** |
|  | **Calibration examiner** | **+** | **+** | **+** | **+** | **+** | **NR** | **NR** | **NR** | **NR** | **NR** | **+** | **NR** | **+** | **NR** | **+** | **+** | **NR** | **NR** | **NR** | **NR** | **NR** |
|  | **Reproducibility data shown** | **+** | **+** | **+** | **-** | **+** | **-** | **-** | **-** | **-** | **-** | **-** | **-** | **-** | **-** | **-** | **-** | **-** | **-** | **-** | **-** | **-** |
| **Authors’ estimated risk of bias** | | **Low** | **Low** | **Low** | **Low** | **Low** | **Moderate** | **Low** | **High** | **Moderate** | **High** | **Low** | **Moderate** | **High** | **Low** | **Low** | **Moderate** | **Moderate** | **Moderate** | **High** | **High** | **High** |

As stated by Van der Weijden et al. (2009) (9), a low risk of bias was defined as presence of random allocation, blinding of both participants and examiners, balanced experimental groups, reporting loss to follow-up, identical treatment between groups (apart from the intervention) and defined eligibility criteria. Absence of one criterion was considered a possible moderate risk of bias. Studies meeting five or less criteria were given the high-risk estimate.

NA: not applicable

NR: not reported

+: study met criterion

-: study did not meet criterion

*: reporting criteria for estimating the potential risk of bias

◊: calculated by the authors of this review based on the data presented in the selected paper

?: unclear; information was insufficient

# Appendix S4: Mean (SD) scores for the different intervention groups with reported significance of changes within groups, including various indices and their modifications extracted from the included papers.

**Appendix S4a (PI): Turesky et al. (1970) modification of the Quigley & Hein Plaque Index (1962).**

| **#** |  |  | **Mean (SD)** | | | | **Significant**  **within groups** |
| --- | --- | --- | --- | --- | --- | --- | --- |
|  | **DF GROUP** | **N** | **Baseline** | **End** | **Difference** | |  |
|  |  |  |  |  | **Mean** | **%** |  |
| Akwagyiram et al. (2018) (37) | BS67%  NaF | (N=118)  (N=122) | 3.04♣(0.206♣◊)  3.00♣(0.210♣◊) | 2.52♣(0.348♣◊)  2.95♣(0.221♣◊) | -0.52◊(?)  -0.05◊(?) | -17,1%◊(?)  -1.7%◊(?) | NR  NR |
| Bosma et al. (2018) (38)  ***Single-brushing*** | **A)** BS67%herbal  **B)** BS67%  **C)** BS62%herbal  **D)** NaF | (N=56)  (N=56)  (N=56)  (N=56) | 2.90(0.308♦)  2.92(0.312♦)  2.89(0.304♦)  2.86(0.309♦) | 1.76♦(0.514♦)  1.78♦(0.492♦)  1.77♦(0.469♦)  1.96♦(0.470) | -1.13♦(0.430♦)  -1.14♦(0.426♦)  -1.11♦(0.419♦)  -0.90♦(0.409♦) | -39.0%◊(?)  -39.0%◊(?)  -38.4%◊(?)  -31.5%◊(?) | NR  NR  NR  NR |
| Jose et al. (2018) (54) | BS67%  NaF | (N=118)  (N=117) | 3.05♦(0.403♦)  3.05♦(0.353♦) | 2.47♦(0.437♦)  2.92♦(0.399♦) | -0.58◊(?)  -0.13◊(?) | -0.19%◊(?)  -4.3%◊(?) | NR  NR |
| Hosadurga et al. (2017) (36) | BS  MFP | (N=25)  (N=20) | 1.99(0.44)  2.07(0.44) | 1.57(0.356)  1.41(0.316) | -0.42♦(0.34♦)  -0.66♦(0.34♦) | -21.08%(?)  -31.85%(?) | YES  YES |
| Mason et al. (2017) (53)  *Sub-study 1*  ***Single-brushing*** | **A)** BS67%  **B)** BS45%  **C)** NaF | (N=60)  (N=60)  (N=60) | 2.54♦(0.50♦)  2.48♦(0.45♦)  2.54♦(0.49♦) | 0.83♦(0.50♦)  0.80♦(0.51♦)  0.99♦(0.57♦) | -1.71(0.36♦)  -1.70(0.36♦)  -1.55(0.36♦) | -67.3%◊(?)  -68.6%◊(?)  -61.0%◊(?) | NR  NR  NR |
| Mason et al. (2017) (53)  *Sub-study 2*  ***Single-brushing*** | BS67%  SnF | (N=54♦)  (N=54♦) | 2.59♦(0.47♦)  2.62♦(0.42♦) | 0.80♦(0.51♦)  1.10♦(0.51♦) | -1.80(0.43♦)  -1.52(0.43♦) | -69.5%◊(?)  -58.0%◊(?) | NR  NR |
| Triratana et al. (2015) (51) | BS  **A)** MFP+NaF  **B)** Tcs | (N=45)  (N=45)  (N=45) | 3.56(0.36)  3.53(0.37)  3.55(0.36) | 3.23(0.35)  3.40(0.39)  1.65(0.45) | -0.33♦(0.46♦)  -0.13♦(0.57♦)  -1.90♦(0.56♦) | -9.0%♦(12.52%♦)  -4.2%♦(16.48%♦)  -53.5%♦(13.58%♦) | YES  NO  YES |
| Ghassemi et al. (2008) (50)  daily brushing | BS  Tcs | (N=105)  (N=102) | 2.90(0.40)  2.90(0.39) | 2.55♦(0.43♦)  2.74♦(0.40♦) | -0.34(0.32)  -0.15(0.24) | -11.8%♦(?)  -5.3%♦(?) | YES  YES |
| Ghassemi et al. (2008) **1**  ***Single-brushing*** *(baseline visit)* | BS  Tcs | (N=105)  (N=102) | 2.90(0.40)  2.90(0.39) | 2.36♦(0.46♦)  2.61♦(0.40♦) | -0.54♦(0.26♦)  -0.28♦(0.18♦) | -18.5%♦(?)  -9.8%♦(?) | YES  YES |
| Ghassemi et al. (2008) **2**  ***Single-brushing*** *(final visit)* | BS  Tcs | (N=105)  (N=102) | 2.55(0.43)  2.74(0.40) | 2.09(0.45)  2.52(0.39) | -0.47(0.21)  -0.23(0.15) | -18.3%♦(?)  -8.2%♦(?) | YES  YES |
| Putt et al. (2008) (49)  *Sub-study 1*  *Single-brushing* | **A)** BS65%  **B)** BS20%  **C)** Tcs | (N=65)  (N=65)  (N=65) | 3.16(0.35)  3.18(0.37)  3.16(0.37) | 2.43♦(0.42♦)  2.55♦(0.48♦)  2.64♦(0.42♦) | -0.73(0.26)  -0.64(0.26)  -0.52(0.20) | -23.1%◊(?)  -20.1%◊(?)  -16.5%◊(?) | YES  YES  YES |
| Putt et al. (2008)  *Sub-study 2*  ***Single-brushing*** | **A)** BS48%  **B)** BS20%  **C)** Tcs  **D)** SnF | (N=66)  (N=66)  (N=66)  (N=66) | 3.05(0.35)  3.02(0.36)  3.03(0.33)  3.04(0.40) | 2.33♦(0.42♦)  2.35♦(0.39♦)  2.45♦(0.35♦)  2.45♦(0.38♦) | -0.72(0.28)  -0.68(0.27)  -0.58(0.25)  -0.59(0.26) | -23.6%◊(?)  -22.5%◊(?)  -19.1%◊(?)  -19.4%◊(?) | YES  YES  YES  YES |
| Putt et al. (2008)  *Sub-study 3*  ***Single-brushing*** | **A)** BS48%  **B)** BS27%  **C)** Tcs  **D)** NaF | (N=64)  (N=64)  (N=64)  (N=64) | 3.14(0.31)  3.15(0.34)  3.12(0.40)  3.13(0.34) | 2.36♦(0.35♦)  2.42♦(0.36♦)  2.51♦(0.35♦)  2.54♦(0.31♦) | -0.78(0.31)  -0.73(0.32)  -0.62(0.27)  -0.58(0.24) | -24.8%◊(?)  -23.2%◊(?)  -19.9%◊(?)  -18.5%◊(?) | YES  YES  YES  YES |
| Putt et al. (2008)  *Sub-study 4*  ***Single-brushing*** | BS65%  NaF | (N=36)  (N=36) | 2.94(0.35)  2.97(0.34) | 2.14♦(0.43♦)  2.50♦(0.36♦) | -0.80(0.31)  -0.47(0.23) | -27.2%◊(?)  -15.8%◊(?) | YES  YES |
| Putt et al. (2008)  *Sub-study 5*  *Single-brushing* | BS20%  Tcs | (N=41)  (N=41) | 2.28(0.36)  2.25(0.33) | 1.21♦(0.50♦)  1.38♦(0.47♦) | -1.07(0.38)  -0.87(0.37) | -46.9%◊(?)  -38.7%◊(?) | YES  YES |
| Ozaki et al. (2006) * (48)  *whole mouth* | BS  Tcs | (N=20)  (N=22) | 2.28♦(0.68♦)  2.26♦(0.60♦) | 1.82♦(0.58♦)  1.89♦(0.57♦) | -0.46♦(0.19♦)  -0.38♦(0.33♦) | -20.6%♦(8.14♦)  -16.5%♦(14.22♦) | YES*  YES* |
| Mullally et al. (1995) (5) | BS  MFP+NaF | *(*N*=*35◊)  *(*N*=*35◊) | 2.4(0.4)  2.6(0.5) | 1.6(0.3)  1.8(0.4) | -0.8(0.3)  -0.8(0.3) | -33.3%◊(?)  -30.8%◊(?) | YES  YES |
| Saxer et al. (1995) (45) | BS  Non-BS | (N=23◊)  (N=23◊) | 2.22(0.44)  2.21(0.50) | 2.19(0.40)  2.15(0.44) | -0.03 ◊(?)  -0.06 ◊(?) | -1%◊(?)  -3%◊(?) | NO  NO |
| Yankell et al. (1993) (42) | BS  Non-BS | (N=60)  (N=63) | 3.08(0.65)  3.18(0.53) | 2.45(0.69)  2.75(0.58) | -0.63 ◊(?)  -0.43 ◊(?) | -20.5%◊(?)  -13.5%◊(?) | YES  NO |
| Emling and Yankell (1988) (41)  ***Single-brushing*** | BS-P DF  NaF-DF | (N=6◊)  (N=6◊) | 2.71(0.83)  2.71(0.85) | 2.13(1.15)  1.82(1.27) | -0.58◊(?)  -0.89◊(?) | -21.4%◊(?)  -32.8%◊(?) | YES  YES |
| Yankell and Emling (1988) (40) | BS  **A)** Non-BS  **B)** NaF | (N=20◊)  (N=20◊)  (N=20◊) | 1.06(0.52)  1.15(0.71)  1.16(0.54) | 0.98(0.69)  1.10(0.44)  0.95(0.72) | -0.08◊(?)  -0.05◊(?)  -0.21◊(?) | -7.5% ◊(?)  -4.3% ◊(?)  -18.1%◊(?) | NO  NO  NO |

* based on pooled data ♦ additional data provided by the original authors ♣ additional data from ClinicalTrials.gov

**Appendix S4b (PI): Distal Mesial Plaque Index Fischman et al. (1987).**

| **#** |  | **Mean (SD)** | | | | **Significant within groups** |
| --- | --- | --- | --- | --- | --- | --- |
|  | **GROUP** | **Baseline** | **End** | **Difference** | |  |
|  |  |  |  | **Mean** | **%** |  |
| Mankodi et al. (1998) (47)  *single-brushing* | BS-DF(N=34◊)  NaF-DF(N=34◊)  MFP+NaF-DF(N=34◊) | NR | NR | NR | -66.93%(?)  -58.39%(?)  -58.65%(?) | NR  NR  NR |

**Appendix S4c (PI):** **Löe (1967) modification of the Silness & Löe (Silness & Löe 1964) Plaque Index.**

| **#** |  | **Mean (SD)** | | | | **Significant within groups** |
| --- | --- | --- | --- | --- | --- | --- |
|  | **GROUP** | **Baseline** | **End** | **Difference** | |  |
|  |  |  |  | **Mean** | **%** |  |
| Beiswanger et al. (1997) (46) | BS-DF(N=147)  **A)** NaF-DF(N=140)  **B)** SnF-DF(N=267) | 0.73(0.36◊)  0.67(0.35◊)  0.73(0.33◊) | 0.58(0.24◊)  0.54(0.24◊)  0.55(0.33◊) | -0.15◊(?)  -0.13◊(?)  -0.18◊(?) | -20.5%◊(?)  -19.4%◊(?)  -24.7%◊(?) | NO  NO  NO |
| Winer et al. (1986) (39) | BS-DF(N=18)  Non-BS DF(N=21) | 2.20◊(1.07◊)  2.04◊(0.93◊) | 1.37◊(0.96◊)  1.21◊(0.76◊) | -0.83◊(?)  -0.83◊(?) | -37.6%(?)  -40.5%(?) | YES  YES |

**Appendix S4d (PI): Podshadley & Haley (1968) Patient Hygiene Performance Index.**

| **#** |  | **Mean (SD)** | | | | **Significant within groups** |
| --- | --- | --- | --- | --- | --- | --- |
|  | **GROUP** | **Baseline** | **End** | **Difference** | |  |
|  |  |  |  | **Mean** | **%** |  |
| Al-Kholani. (2011) (35) | BS-DF(N=16)  **A)** Non-BS Herbal DF(N=16)  **B)** NaF-DF(N=16) | 2.56(0.06)  2.57(0.09)  2.71(0.12) | 0.96(0.07)  1.07(0.06)  1.77(0.06) | -1.60◊(?)  -1.50◊(?)  -0.94◊(?) | -61.2%(?)  -57.2%(?)  -33.3%(?) | YES  YES  NO |

**Appendix S4e (PI): Lange et al. (1977) Approximal Plaque Index.**

| **#** |  | **Mean (SD)** | | | | **Significant within groups** |
| --- | --- | --- | --- | --- | --- | --- |
|  | **GROUP** | **Baseline** | **End** | **Difference** | |  |
|  |  |  |  | **Mean** | **%** |  |
| Al-Kholani. (2011) (35) | BS-DF(N=16)  **A)** Non-BS Herbal DF(N=16)  **B)** NaF-DF(N=16) | 65.3 (2.1)  63.5 (1.7)  61.2 (2.2) | 26.1 (1.1)  28.3 (1.2)  45.6 (1.5) | -39.2◊(?)  -35.2◊(?)  -15.6◊(?) | -57.4%(?)  -52.8%(?)  -23.0%(?) | YES  YES  NO |

**Appendix S4f (GI):** **Löe & Silness (1963) and Löe (1967) Gingival Index.**

| **#** |  | **Mean (SD)** | | | | **Significant within groups** |
| --- | --- | --- | --- | --- | --- | --- |
|  | **GROUP** | **Baseline** | **End** | **Difference** | |  |
|  |  |  |  | **Mean** | **%** |  |
| Hosadurga et al. (2017)^1^ (36) | BS-DF(N=25)  MFP-DF(N=20) | 1.468(0.329)  1.493((0.327) | 1.088(0.289)  1.207(0.114) | -0.38♦(0.35♦)  -0.29♦(0.34♦) | -25.92%(?)  -19.14%(?) | YES  YES |
| Triratana et al. (2015)^1^ (51) | BS-P DF(N=45)  **A)** MFP+NaF-DF(N=45)  **B)** Tcs-DF(N=45) | 1.77(0.20)  1.77(0.15)  1.78(0.20) | 1.47(0.30)  1.57(0.28)  0.95(0.26) | -0.30♦(0.34♦)  -0,20♦(0.35♦)  -0.83♦(0.22♦) | -17.4%(18.60%♦)  -11.8%(19.06%♦)  -46.6%(12.01%♦) | YES  YES  YES |
| Al-Kholani. (2011)^1^ (35) | BS-DF(N=16)  **A)** Non-BS-Herbal DF(N=16)  **B)** NaF DF (N=16) | 1.86 (0.11)  1.75 (0.08)  1.87 (0.11) | 0.57 (0.04)  0.49 (0.03)  1.09 (0.9) | -1.29◊(?)  -1.26◊(?)  -0.78◊(?) | -68.0%(?)  -70.6%(?)  -40.5%(?) | YES  YES  NO |
| Ozaki et al. (2006) *^1^ (48) | BS-DF(N=20)  Tcs-DF(N=22) | 1.10♦(0.25♦)  1.11♦(0.27♦) | 0.82♦(0.35♦)  0.73♦(0.27♦) | -0.28♦(0.21♦)  -0.38♦(0.13♦) | -27.2%♦(20.83%♦)  -35.7%♦(9.11%♦) | YES*  YES* |
| Beiswanger et al. (1997)^2^ (46) | BS-DF(N=147)  **A)** NaF-DF(N=140)  **B)** SnF-DF(N=267) | 0.89(0.24◊)  0.84(0.24◊)  0.86(0.33◊) | 0.74(0.24◊)  0.78(0.24◊)  0.64(0.16◊) | -0.15(?)  -0.06(?)  -0.22(?) | -16.7%◊(?)  -7.1%◊(?)  -25.6%◊(?) | NO  NO  NO |
| Mullally et al. (1995)^1^ (5) | BS-DF(N=35◊)  MFP+NaF-DF(N=35◊) | 0.86(0.09)  0.86(0.08) | 0.41(0.19)  0.47(0.17) | -0.45(0.17)  -0.39(0.17) | -52.3%◊(?)  -45.3%◊(?) | YES  YES |
| Saxer et al. (1995)^1^ (45) | BS-DF (N=23◊)  Non-BS Herbal-DF(N=23◊) | 1.58(0.62)  1.46(0.58) | 0.89(0.30)  0.87(0.35) | -0.69 ◊(?)  -0.59◊(?) | -44%(?)  -40%(?) | YES  YES |
| Yankell et al. (1993) *^1^ (42) | BS-DF(N=60)  Non-BS Herbal-DF(N=63) | 2.10◊(0.15◊)  2.11◊(0.17◊) | 0.84◊(0.64◊)  0.84◊(0.48◊) | -1.26◊(?)  -1.27◊(?) | -60.0%◊(?)  -60.2%◊(?) | YES*  YES* |
| Yankell and Emling (1988)^1^ (40) | BS-DF(N=20◊)  **A)** Non-BS DF (N=20◊)  **B)** NaF-DF(N=20◊) | 0.56(0.25)  0.53(0.25)  0.51(0.26) | 0.51(0.27)  0.56(0.31)  0.55(0.25) | -0.05◊(?)  +0.03◊(?)  +0.04◊(?) | -8.9% ◊(?)  +5.7% ◊(?)  +7.8%◊(?) | NO  NO  YES |
| Winer et al. (1986) *^1,2^ (39) | BS-DF(N=18 )  Non-BS DF(N=21) | 2.95◊(0.49◊)  2.87◊ (0.64◊) | 2.56◊ (0.59◊)  2.61◊ (0.73◊) | -0.40◊(?)  -0.26◊(?) | -13.5%(?)  -9.1%(?) | YES  YES |

* based on pooled data

^1^ Löe & Silness Gingival Index (1963)

^2^ Löe Gingival Index (1967)

**Appendix S4g (GI): Modified Gingival Index (MGI). Gingival Index of Löe & Silness (1963) modified by Lobene et al. (1986).**

| **#** |  | **Mean (SD)** | | | | **Significant within groups** |
| --- | --- | --- | --- | --- | --- | --- |
|  | **GROUP** | **Baseline** | **End** | **Difference** | |  |
|  |  |  |  | **Mean** | **%** |  |
| Akwagyiram et al. (2018) (37) | BS67%(N=118)  NaF(N=122) | 2.53♣(0.185♣◊)  2.54♣(0.199♣◊) | 2.23♣(0.228♣◊)  2.43♣(0.228♣◊) | -0.30◊(?)  -0.11◊(?) | -11.86%◊(?)  -4,3%◊(?) | NR  NR |
| Jose et al. (2018) (54) | BS67%(N=118)  NaF(N=117) | 2.32♦(0.345♦)  2.35♦(0.327♦) | 1.26♦(0.505♦)  1.93♦(0.408♦) | -1.06◊(?)  -0.42◊(?) | -45.7%◊(?)  -17.9%◊(?) | NR  NR |
| Lomax et al. (2016) (52) | BS-DF(N=66)  NaF-DF(N=69) | 1.26(0.28)  1.26(0.23) | 0.64(0.24)  0.91(0.27) | -0.61(0.14)  -0.34(0.16) | -48.4%◊(?)  -27.0%◊(?) | NR  NR |

**Appendix S4h-1 (BI): Ainamo & Bay (1975) Gingival Bleeding Index.**

| **#** |  | **Mean (SD)** | | | | **Significant within groups** |
| --- | --- | --- | --- | --- | --- | --- |
|  | **GROUP** | **Baseline** | **End** | **Difference** | |  |
|  |  |  |  | **Mean** | **%** |  |
| Mullally et al. (1995)**^1^** (5) | BS-DF(N=35◊)  MFP+NaF-DF(N=35◊) | NR | NR | NR | -32.3(14.6)  -29.1(13.4) | YES  NO |
| Taller (1993)**^2^** (43) | BS-DF(N=14 )  Non-BS DF(N=13) | 0.57◊(0.16◊)  0.57◊(0.07◊) | 0.51◊(0.18◊)  0.52◊(0.09◊) | -0.06(0.20)  -0.05(0.13) | -11%(?)  -9%(?) | NO  NO |

**^1^** Bleeding points on four areas of 12 teeth (17, 15, 13, 21, 24, 26, 37, 35, 33, 42, 44, 46); % of sites bleeding calculated for each participant.

**^2^** Bleeding (Ainamo & Bay Gingival Bleeding Index (1975) points scores (yes/no) on four areas of six teeth (16, 21, 24, 36, 41, 44) totaled per participant.

**Appendix S4h-2 (BI): Abrams, Caton and Polson (1984) Bleeding on Probing Index.**

| **#** |  | **Mean (SD)** | | | | **Significant within groups** |
| --- | --- | --- | --- | --- | --- | --- |
|  | **GROUP** | **Baseline** | **End** | **Difference** | |  |
|  |  |  |  | **Mean** | **%** |  |
| Yankell et al. (1993) (42) | BS-DF(N=60)  Non-BS Herbal-DF(N=63) | 0.26(0.25)  0.31(0.23) | 0.24(0.27)  0.36(0.32) | -0.02 ◊(?)  +0.05 ◊(?) | -7.7%◊(?)  +16.1%◊(?) | NO  YES |
| Yankell and Emling (1988) (40) | BS-DF (N=20◊)  **A)** Non-BS DF (N=20◊)  **B)** NaF-DF (N=20◊) | 0.50(0.22)  0.44(0.26)  0.31(0.23) | 0.26(0.20)  0.44(0.28)  0.34(0.33) | -0.24◊(?)  0,0◊(?)  +0.03◊(?) | -48.0% ◊(?)  0,0%◊(?)  +9.7%◊(?) | YES  NO  NO |

**Appendix S4i-1 (BI):** **Ainamo & Bay Gingival Bleeding Index (1975) and the modification by Saxer et al. (1977).**

| **#** |  | **Mean (SD)** | | | | **Significant within groups** |
| --- | --- | --- | --- | --- | --- | --- |
|  | **GROUP** | **Baseline** | **End** | **Difference** | |  |
|  |  |  |  | **Mean** | **%** |  |
| Saxer et al. (1995)**^1^** (45) | BS-DF(N=23◊)  Non-BS Herbal-DF(N=23◊) | 1.19(0.46) 1.07(0.43) | 0.73(0.32)  0.64(0.27) | -0.46◊(?)  -0.43◊(?) | -39%(?)  -40%(?) | YES  YES |
| Saxer et al. (1994)**^1^** (44) | BS-DF(N=11◊)  Non-BS F-DF(N=11◊) | 1.06◊(0.28◊)  1.03◊(0.26◊) | 0.83◊(0.31◊)  1.08◊(0.38◊) | -5.60◊(?)  +1.25◊(?) | -22.0%◊(?)  -5.1%◊(?) | YES  NO |

**^1^** Bleeding points on four areas of 12 teeth (17, 15, 13, 21, 24, 26, 37, 35, 33, 42, 44, 46); % of sites bleeding calculated for each participant.

**Appendix S4i-2 (BI):** **Gingival Bleeding Index by Saxton & van der Ouderaa (1989).**

| **#** |  | **Mean (SD)** | | | | **Significant within groups** |
| --- | --- | --- | --- | --- | --- | --- |
|  | **GROUP** | **Baseline** | **End** | **Difference** | |  |
|  |  |  |  | **Mean** | **%** |  |
| Akwagyiram et al. (2018) (37) | BS67%(N=118)  NaF(N=122) | 0.45♣(0.152♣◊)  0.47♣(0.155♣◊) | 0.19♣(0.130♣◊)  0.37♣(0.155♣◊) | -0.26◊(?)  -0.10◊(?) | -57.8%◊(?)  -21,3%◊(?) | NR  NR |
| Jose et al. (2018) (54) | BS67%(N=118)  NaF(N=117) | 0.38♦(0.167♦)  0.37♦(0.170♦) | 0.17♦(0.116♦)  0.31♦(0.159♦) | -0.21◊(?)  -0.06◊(?) | -55.3%◊(?)  -16.2%◊(?) | NR  NR |
| Lomax et al. (2016) (52) | BS-DF(N=66)  NaF-DF(N=69) | 1.00(0.27)  0.94(0.22) | 0.36(0.16)  0.47(0.16) | -0.64(0.18)  -0.48(0.13) | -64.0%◊(?)  -51.1%◊(?) | NR  NR |

**Appendix S4j (BI): Mühlemann & Son (1971) Sulcus Bleeding Index.**

| **#** |  | **Mean (SD)** | | | | **Significant within groups** |
| --- | --- | --- | --- | --- | --- | --- |
|  | **GROUP** | **Baseline** | **End** | **Difference** | |  |
|  |  |  |  | **Mean** | **%** |  |
| Al-Kholani (2011) (35) | BS-DF(N=16)  **A)** Non-BS Herbal DF(N=16)  **B)** NaF-DF (N=16) | 45.2 (3.2)  46.4 (2.5)  41.1 (3.4) | 9.1 (1.2)  8.3 (1.0)  22.8 (2.1) | -36.1◊(?)  -38.1◊(?)  -18.3◊(?) | -79.5%(?)  -81.2%(?)  -42.1%(?) | NO  NO  NO |

**Appendix S4k (BI): Number of Bleeding Sites.**

| **#** |  | **Mean (SD)** | | | | **Significant within groups** |
| --- | --- | --- | --- | --- | --- | --- |
|  | **GROUP** | **Baseline** | **End** | **Difference** | |  |
|  |  |  |  | **Mean** | **%** |  |
| Akwagyiram et al. (2018) (37) | BS67%  NaF | 45.13(1.393)  45.84(1.448) | 18.67(1.142)  36.39(1.510) | -26.46◊(?)  -9.45◊(?) | -58.6%◊(?)  -20.6%◊(?) | NR  NR |
| Jose et al. (2018) (54) | BS67%(N=118)  NaF(N=117) | 29.76♣(8.238♣)  29.05♣(8.390♣) | 13.24♣(7.663♣)  24.90♣(9.044♣) | -16.52◊(?)  -4.15◊(?) | -55.5%◊(?)  -14.3%◊(?) | NR  NR |
| Lomax et al. (2016) (52) | BS-DF(N=66)  NaF-DF(N=69) | 76.0(13.06)  73.5(12.25) | 33.2(12.21)  42.5(12.85) | -42.7(10.15)  -31.0(8.71) | -56.2%◊(?)  -42.2%◊(?) | NR  NR |
| Beiswanger et al. (1997) (46) | BS-DF(N=147)  **A)** NaF-DF(N=140)  **B)** SnF-DF (N=267) | 27.42(21.95◊)  23.36(16.21◊)  24.87(17.16◊) | 21.82(10.67◊)  22.25(10.65◊)  16.13(10.62◊) | -5.6◊(?)  -1.11◊(?)  -8.74◊(?) | -20.4%◊(?)  -4.8%◊(?)  -35.1%◊(?) | NO  NO  YES |
| Saxer et al. (1994) (44) | BS-DF(N=11◊)  Non-BS F-DF (N=11◊) | 21.30 (3.77)  20.33 (3.73) | 17.10 (5.63)  20.42 (4.08) | -4.2◊(?)  +0.08 (?) | -19.7%◊(?)  +0.4%◊(?) | YES  NO |

# Appendix S5: Overview of all reported indices included in this systematic review.

| Plaque indices | Gingival indices | Bleeding indices |
| --- | --- | --- |
| The Turesky et al. (1970) modification of the Quigley and Hein Plaque Index (1962) (TQ&H) | The Löe & Silness Gingival Index (1963) | The Ainamo & Bay Gingival Bleeding Index (1975) |
| The Fischman et al. (1987) Distal Mesial Plaque Index | The Löe & Silness Gingival Index (1967) | The Saxer et al. (1977) Papillary bleeding Index modification of the Ainamo & Bay Bleeding Index (1975) |
| The Löe. (1967) modification of the Silness & Löe Plaque Index (1964) | The Lobene et al. (1986) modification of the Gingival Index (MGI) | The Saxton & Van der Ouderaa (1989) Gingival Bleeding Index |
| The Podshadley & Haley Patient Hygiene Performance Index (1968) |  | The Abrams, Caton and Polson Bleeding 0n Probing Index (1984) |
| The Lange et al. Approximal Plaque Index (1977) |  | The Mühlemann & Son Sulcus Bleeding Index (1971). |

**Appendix S6: PI**

**Forest plots of BASELINE, END and DIFFERENCE PLAQUE scores for the comparisons of dentifrices containing BS with negative and positive controls without BS.**

**Appendix S6-A) (PI): Turesky et al. (1970) modification of the Quigley & Hein (1962) Plaque Index analysing the single-brushing experiments.**

**Appendix S6-A1)** **Forest plot of the BASELINE PLAQUE scores of the SINGLE-BRUSHING exercises analysing the Turesky et al. (1970) modification of the Quigley and Hein (1962) Plaque Index for the comparison of dentifrices with BS and NEGATIVE control dentifrices without BS;** no significant difference was observed between groups.

**Appendix S6-A2) Forest plot of the BASELINE PLAQUE scores of the SINGLE-BRUSHING exercises analysing the Turesky et al. (1970) modification of the Quigley and Hein (1962) Plaque Index for the comparison of dentifrices with BS and POSITIVE control dentifrices without BS;** no significant difference was observed between groups.


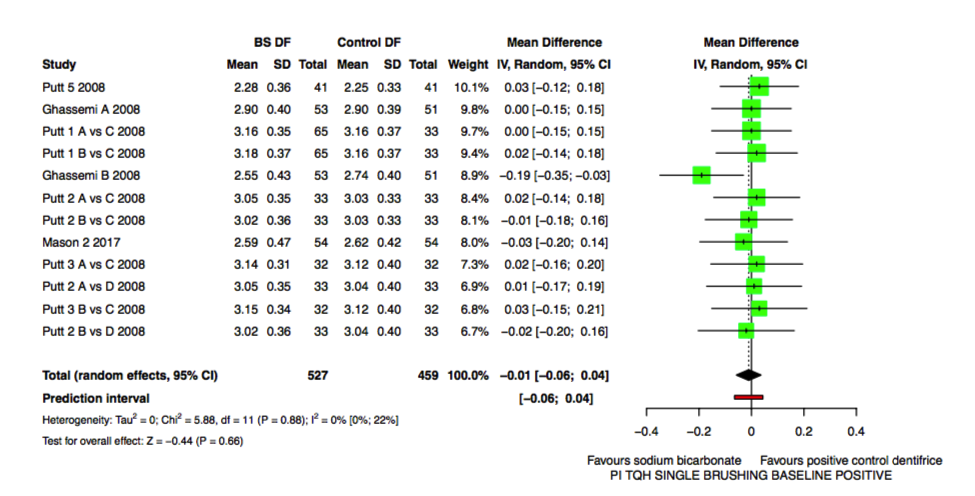
**Appendix S6-A3) Forest plot of the END PLAQUE scores of the SINGLE-BRUSHING exercises analysing the Turesky et al. (1970) modification of the Quigley and Hein (1962) Plaque Index for the comparison of dentifrices with BS and NEGATIVE control dentifrices without BS;** a significant difference was observed in favour of the use of BS.

**Appendix S6-A4) Forest plot of the END PLAQUE scores of the SINGLE-BRUSHING exercises analysing the Turesky et al. (1970) modification of the Quigley and Hein (1962) Plaque Index for the comparison of dentifrices with BS and POSITIVE control dentifrices without BS;** a significant difference was observed in favour of the use of BS.

**
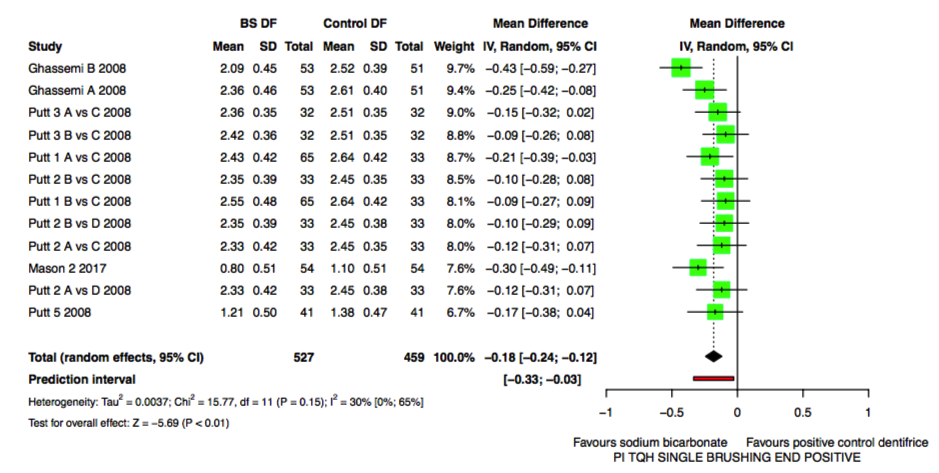
**

**Appendix S6-A5) Forest plot of the DIFFERENCE PLAQUE scores of the SINGLE-BRUSHING exercises analysing the Turesky et al. (1970) modification of the Quigley and Hein (1962) Plaque Index for the comparison of dentifrices with BS and NEGATIVE control dentifrices without BS;** a significant difference was observed between groups.

**Appendix S6-A6) Forest plot of the DIFFERENCE PLAQUE scores of the SINGLE-BRUSHING exercises analysing the Turesky et al. (1970) modification of the Quigley and Hein (1962) Plaque Index for the comparison of dentifrices with BS and POSITIVE control dentifrices without BS;** a significant difference was observed between groups.

**
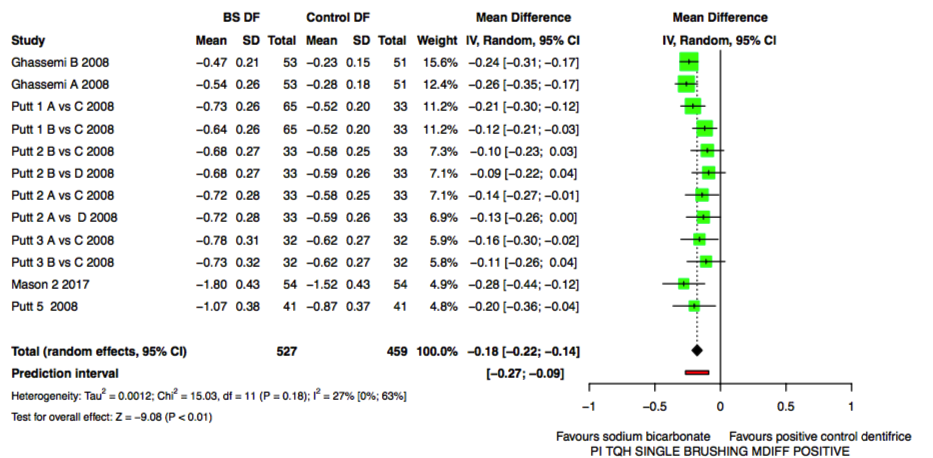
**

**Appendix S6-B) (PI): Turesky et al. (1970) modification of the Quigley & Hein (1962) Plaque Index analysing the FOLLOW-UP brushing experiments.**

**Appendix S6-B1) Forest plot of the BASELINE PLAQUE scores of the FOLLOW-UP brushing exercises analysing the Turesky et al. (1970) modification of the Quigley and Hein (1962) Plaque Index for the comparison of dentifrices with BS and NEGATIVE control dentifrices without BS;** no significant difference was observed between groups.

**Appendix S6-B2) Forest plot of the BASELINE PLAQUE scores of the FOLLOW-UP brushing exercises analysing the Turesky et al. (1970) modification of the Quigley and Hein (1962) Plaque Index for the comparison of dentifrices with BS and POSITIVE control dentifrices without BS;** no significant difference was observed between groups.


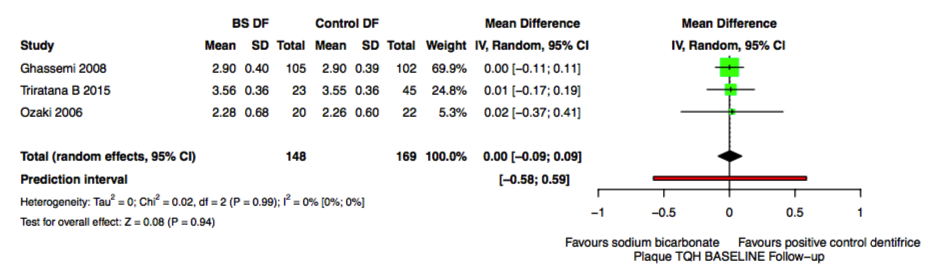


**Appendix S6-B3)** **Forest plot of the meta-subanalysis on ingredients showing END PLAQUE scores of the FOLLOW-UP brushing exercises analysing the Turesky et al. (1970) modification of the Quigley and Hein (1962) Plaque Index for the comparison of dentifrices with BS and NEGATIVE control dentifrices without BS;** a significant difference was observed between groups.

**Appendix S6-B4) Forest plot of the END PLAQUE scores of the FOLLOW-UP brushing exercises analysing the Turesky et al. (1970) modification of the Quigley and Hein (1962) Plaque Index for the comparison of dentifrices with BS and POSITIVE control dentifrices without BS;** no significant difference was observed between groups.


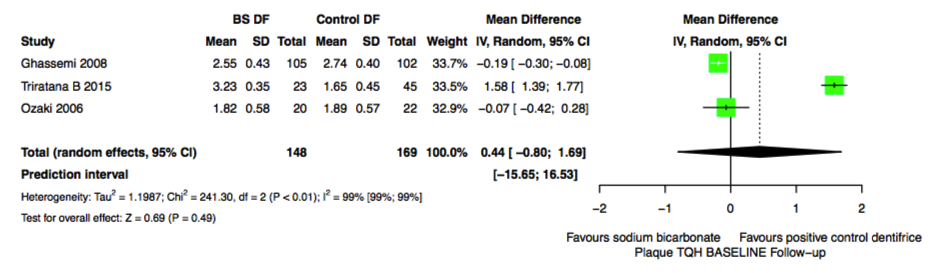


**Appendix S6-B5) Forest plot of the DIFFERENCE PLAQUE scores of the FOLLOW-UP brushing exercises analysing the Turesky et al. (1970) modification of the Quigley and Hein (1962) Plaque Index for the comparison of dentifrices with BS and NEGATIVE control dentifrices without BS;** no significant difference was observed between groups.

**
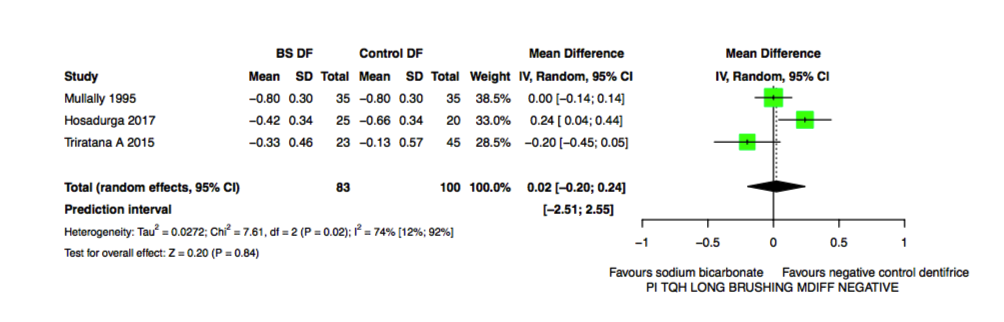
**

**Appendix S6-B6) Forest plot of the DIFFERENCE PLAQUE scores of the FOLLOW-UP brushing exercises analysing the Turesky et al. (1970) modification of the Quigley and Hein (1962) Plaque Index for the comparison of dentifrices with BS and POSITIVE control dentifrices without BS;** no significant difference was observed between groups.


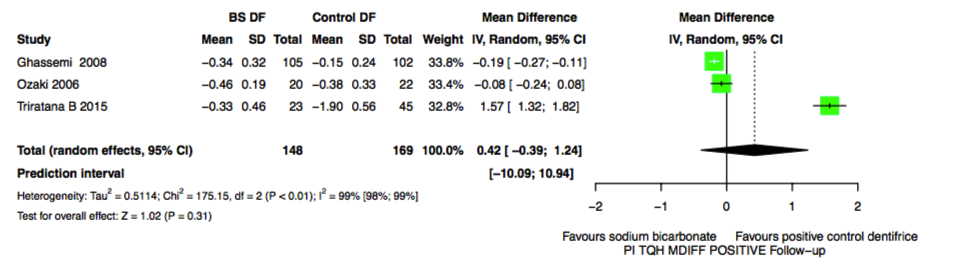


**Appendix S6-C) (PI): The modification by Löe (1967) of the Silness & Löe (Silness & Löe 1964) Plaque Index.**

**Appendix S6-C1) Forest plot of the BASELINE PLAQUE scores of the FOLLOW-UP brushing exercises analysing the modification by Löe (1967) of the Silness & Löe (Silness & Löe 1964) Plaque Index for the comparison of dentifrices with BS and NEGATIVE control dentifrices without BS;** no significant difference was observed between groups.


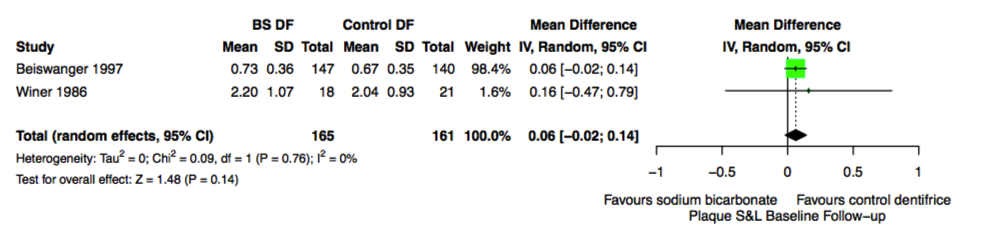


**Appendix S6-C2) Forest plot of the END PLAQUE scores of the FOLLOW-UP brushing exercises analysing the modification by Löe (1967) of the Silness & Löe (Silness & Löe 1964) Plaque Index for the comparison of dentifrices with BS and NEGATIVE control dentifrices without BS;** no significant difference was observed between groups.


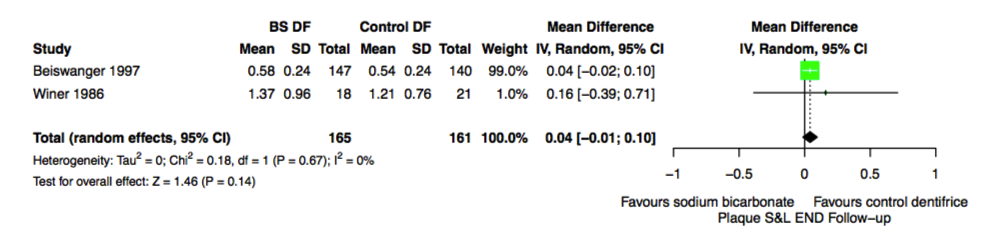


**Appendix S7: GI**

**Forest plots of BASELINE, END and DIFFERENCE gingival index scores for the comparisons of dentifrices containing BS with negative and positive controls without BS.**

**(GI): The Löe & Silness (1963) Gingival Index and the modification of this index by Löe (1967).**

**Appendix S7-1) Forest plot of the BASELINE GINGIVAL index scores of the FOLLOW-UP brushing exercises analysing the Löe & Silness (1963) Gingival Index and the modification of this index by Löe (1967) for the comparison of dentifrices with BS and NEGATIVE control dentifrices without BS;** no significant difference was observed between groups.


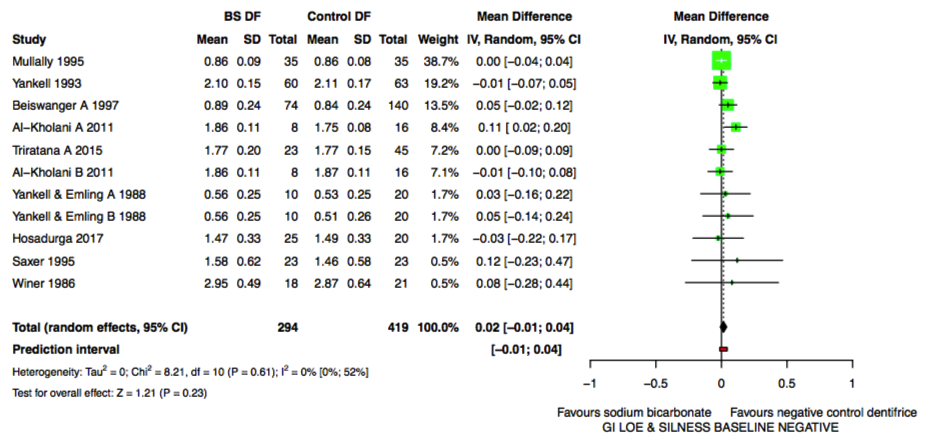


**Appendix S7-2) Forest plot of the BASELINE GINGIVAL index scores of the FOLLOW-UP brushing exercises analysing the Löe & Silness (1963) Gingival Index and the modification of this index by Löe (1967) for the comparison of dentifrices with BS and POSITIVE control dentifrices without BS;** no significant difference was observed between groups.


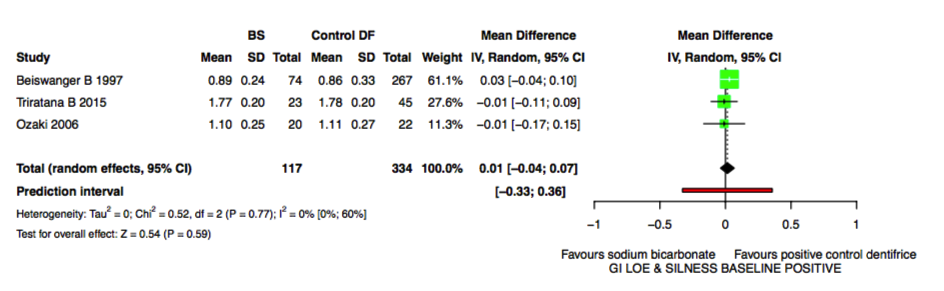


**Appendix S7-3)** **Forest plot of the meta-subanalysis on ingredients showing END** **GINGIVAL index scores of the FOLLOW-UP brushing exercises analysing the Löe & Silness (1963) Gingival Index and the modification of this Index by Löe (1967) for the comparison of dentifrices with BS and NEGATIVE control dentifrices without BS;** no significant difference was observed between groups.

**
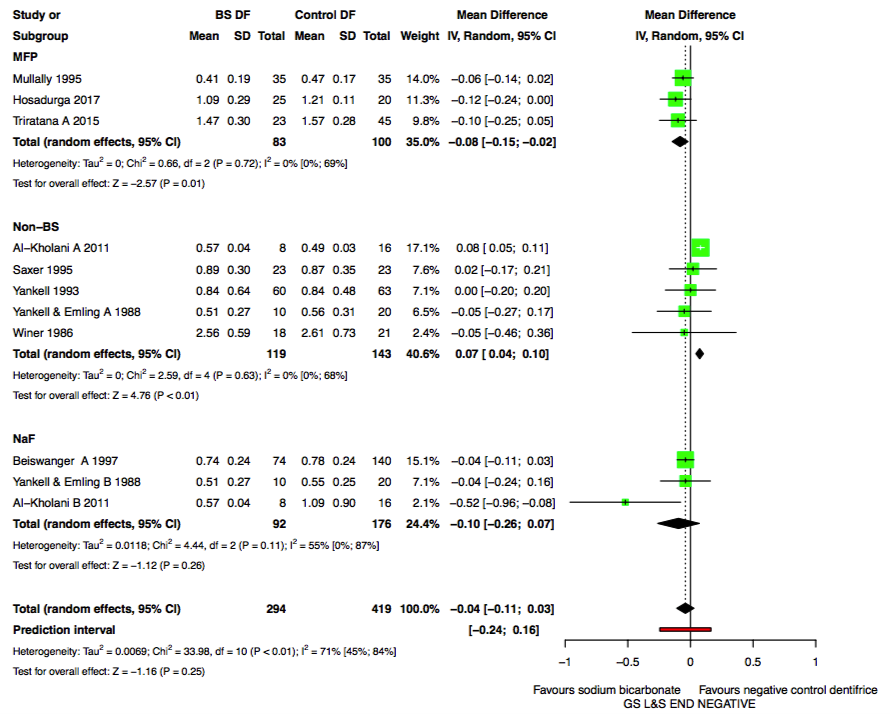
**

**Appendix S7-4) Forest plot of the meta-subanalysis on ingredients showing END GINGIVAL index scores of the FOLLOW-UP brushing exercises analysing the Löe & Silness (1963) Gingival Index and the modification of this index by Löe (1967) for the comparison of dentifrices with BS and POSITIVE control dentifrices without BS;** no significant difference was observed between groups.


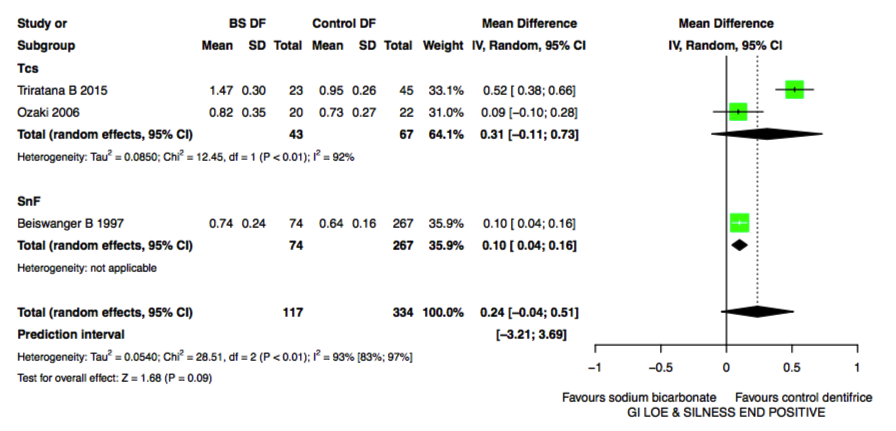


**Appendix S7-5) Forest plot of the DIFFERENCE GINGIVAL index scores of the FOLLOW-UP brushing exercises analysing the Löe & Silness (1963) Gingival Index and the modification of this index by Löe (1967) for the comparison of dentifrices with BS and NEGATIVE control dentifrices without BS;** a significant difference was observed between groups.

**
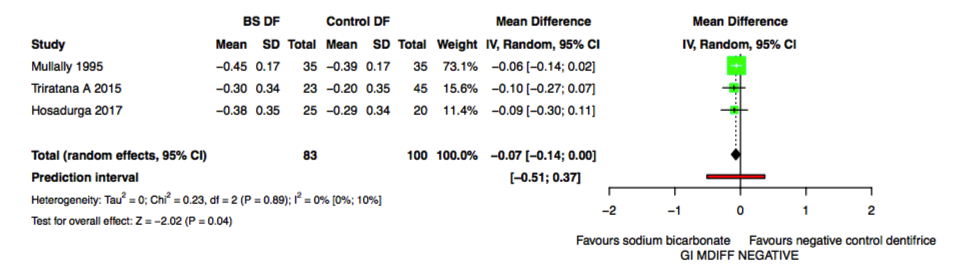
**

**Appendix S7-6)** **Forest plot of the DIFFERENCE GINGIVAL index scores of the FOLLOW-UP brushing exercises analysing the Löe & Silness (1963) Gingival Index and the modification of this index by Löe (1967) for the comparison of dentifrices with BS and POSITIVE control dentifrices without BS;** no significant difference was observed between groups.


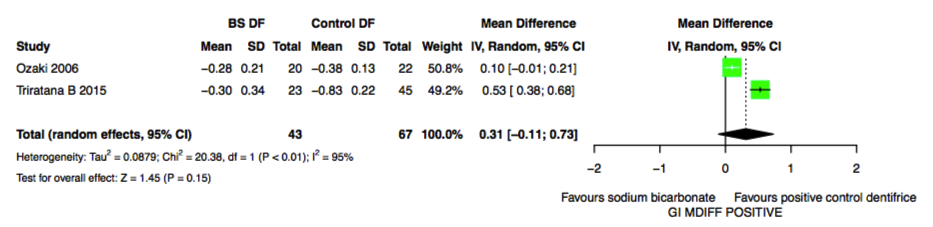


**Appendix S7-7A) Forest plot of the BASELINE GINGIVAL index scores of the FOLLOW-UP brushing exercises analysing the Lobene et al. (1986) modification of the Gingival Index (MGI) for the comparison of dentifrices with BS and NEGATIVE control dentifrices without BS;** a significant difference was observed between groups.

**Appendix S7-7B) Forest plot of the END GINGIVAL index scores of the FOLLOW-UP brushing exercises analysing the Lobene et al. (1986) modification of the Gingival Index (MGI) for the comparison of dentifrices with BS and NEGATIVE control dentifrices without BS;** a significant difference was observed between groups.

**Appendix S8: BI**

**Forest plots of BASELINE and END BLEEDING scores for the comparisons of dentifrices containing BS with negative controls without BS.**

**Appendix S8-A) (BI): The Ainamo & Bay (1975) Gingival Bleeding Index and the Abrams, Caton and Polson (1984) Bleeding on Probing Index.**

**Appendix S8-A1) Forest plot of the BASELINE BLEEDING scores of the FOLLOW-UP brushing exercises analysing The Ainamo & Bay (1975) Gingival Bleeding Index (****See Appendix S4h-1) and the Abrams, Caton and Polson (1984) Bleeding on Probing Index (See Appendix S4h-2) for the comparison of dentifrices with BS and NEGATIVE control dentifrices without BS;** no significant difference was observed between groups.


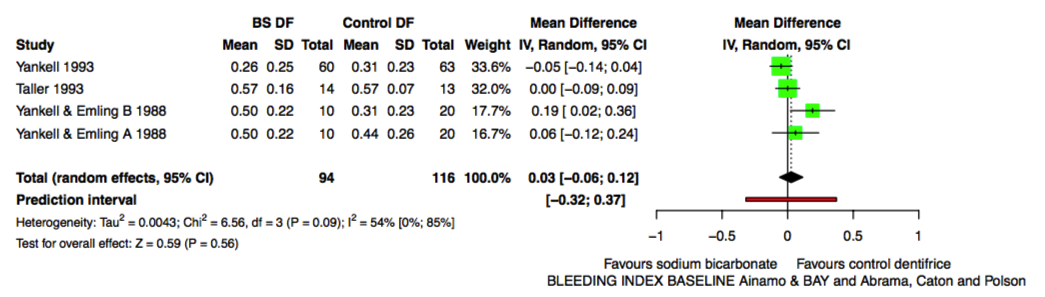


**Appendix S8-A2) Forest plot of the END** **BLEEDING scores of the FOLLOW-UP brushing exercises analysing The Ainamo & Bay (1975) Gingival Bleeding Index (See Appendix S4h-1) and the Abrams, Caton and Polson (1984) Bleeding on Probing Index (65) (See Appendix S4h-2) for the comparison of dentifrices with BS and NEGATIVE control dentifrices without BS;** a significant difference was observed between groups**.**


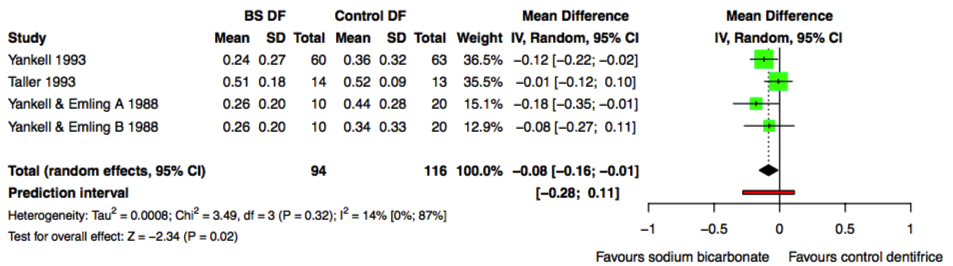


**Appendix S8-B) (BI): The modification by Saxer et al. (1977) of the Ainamo & Bay (1975) Gingival Bleeding Index and the Gingival Bleeding Index by Saxton & van der Ouderaa (1989).**

**Appendix S8-B1) Forest plot of the BASELINE BLEEDING scores of the FOLLOW-UP brushing exercises analysing the modification by Saxer et al. (1977) of the Ainamo & Bay (1975) Gingival Bleeding Index (See Appendix S4i-1) and the Gingival Bleeding Index by Saxton & van der Ouderaa (1989) (See Appendix S4i-2) for the comparison of dentifrices with BS and NEGATIVE control dentifrices without BS;** no significant difference was observed between groups.

**Appendix S8-B2) Forest plot of the END BLEEDING scores of the FOLLOW-UP brushing exercises analysing the modification by Saxer et al. (1977) of the Ainamo & Bay (1975) Gingival Bleeding Index (See Appendix S4i-1) and the Gingival Bleeding Index by Saxton & van der Ouderaa (1989) (See Appendix S4i-2) for the comparison of dentifrices with BS and NEGATIVE control dentifrices without BS;** a significant difference was observed between groups.

**Appendix S9.**

**S9-A) 3. Contour-enhanced funnel plot with trimfill of the meta-analysis showing BASELINE PLAQUE scores of the SINGLE-BRUSHING exercises analysing the Turesky et al. (1970) modification of the Quigley and Hein (1962) Plaque Index for the comparison of dentifrices with BS and POSITIVE control dentifrices without BS.** Egger’s test shows a non-significant p-value (p= 0.85) *.

**
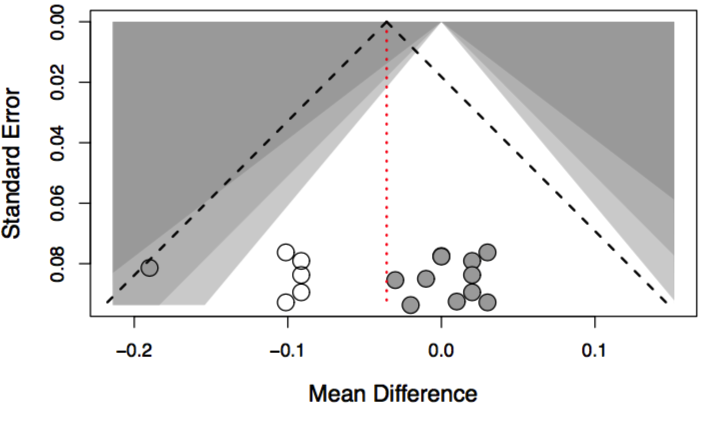
**


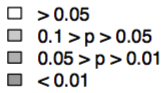


*A test for funnel plot asymmetry (sometimes referred to as a test for small study effects) examines whether the association between estimated intervention effects and a measure of study size is greater than might be expected to occur by chance. These tests typically have low power, so even when a test does not provide evidence of asymmetry, bias cannot be excluded (71).

The Counter- Enhanced Funnel plot is an enhancement to the usual funnel plot proposed to allow considering the statistical significance of study estimates (70). The above funnel is centered at the model estimate. Color of line represent random effects estimate. The filled circles represent estimated treatment effect (mean difference) and its precision (standard error) for each individual study. Contour lines representing well established levels of statistical significance are added to the funnel plot to indicate regions where a test of treatment effect is significant. Contour lines indicating conventional milestones in levels of statistical significance (e.g., <0.01, <0.05, <0.1) are added (73). P-values correspond to a trial’s treatment effect (72). The unshaded (i.e., white) region in the middle corresponds to non-significant results, the medium gray region to significant results at the 5% level and the dark gray region to the 1% level.

The open circles are missing studies filled in by a Trim-and-Filled Method. The basic idea of the trim-and-fill method is to add studies to the funnel plot until it becomes symmetric (73). The counter-funnel plot could be used naturally in conjunction with the trim-and-fill method because the latter informs the likely location of missing studies (70).

Visually, shows the funnel plot asymmetry. However, Egger’s test for asymmetry is not significant (*p*=0.85), indicating a publication bias mechanism is not a major cause for concern here (71).

**Appendix** **S9-B)**

**S9-B)** **Contour-enhanced funnel plot with trimfill of the meta-analysis showing END PLAQUE scores of the SINGLE-BRUSHING exercises analysing the Turesky et al. (1970) modification of the Quigley and Hein (1962) Plaque Index for the comparison of dentifrices with BS and POSITIVE control dentifrices without BS.** Egger’s test shows a non-significant *p*-value (*p*= 0.08).


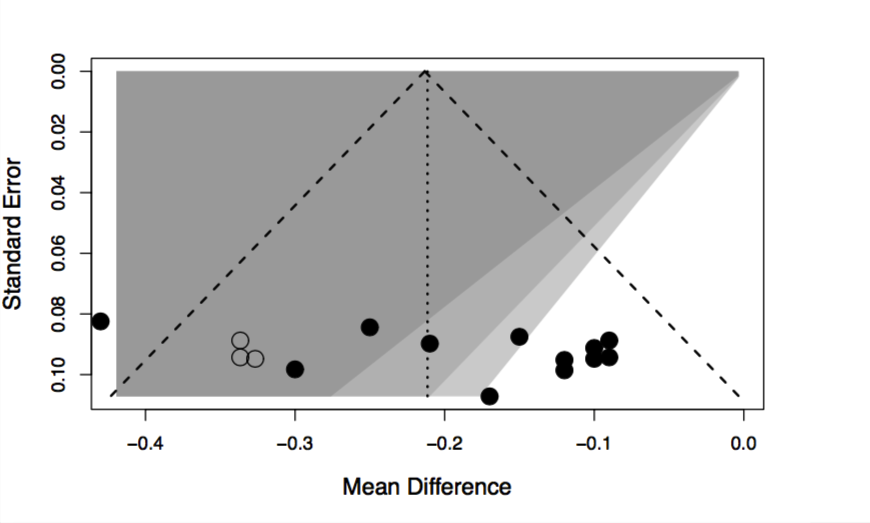


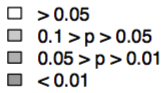


The Counter- Enhanced Funnel plot is an enhancement to the usual funnel plot proposed to allow considering the statistical significance of study estimates (70). The above funnel is centered at the model estimate. Color of line represent random effects estimate. The filled circles represent estimated treatment effect (mean difference) and its precision (standard error) for each individual study. Contour lines representing well established levels of statistical significance are added to the funnel plot to indicate regions where a test of treatment effect is significant. Contour lines indicating conventional milestones in levels of statistical significance (e.g., <0.01, <0.05, <0.1) are added. The unshaded (i.e., white) region in the middle corresponds to *p*-values greater than .10, the light gray-shaded region corresponds to *p*-values between .10 and .05, the gray-shaded region corresponds to p-values between .05 and .01, and the region outside of the funnel corresponds to *p*-values below.

The open circles are missing comparisons filled in by a Trim-and-Filled Method. The basic idea of the trim-and-fill method is to add studies to the funnel plot until it becomes symmetric (73). The counter-funnel plot could be used naturally in conjunction with the trim-and-fill method because the latter informs the likely location of missing studies (70).

Visually, shows the funnel plot minor asymmetry. However, Egger’s test for asymmetry is not significant (*p*=0.08), indicating a publication bias mechanism is not a major cause for concern here (71). Also, the contour-enhanced funnel plot shows that the “missing” studies are expected to lie in areas of high statistical significance (whereas the majority of available studies are nonsignificant), indicating that the observed asymmetry may not be due to publication bias based on statistical significance (70).

**Appendix S10.**

**S10-A) A Contour-enhanced funnel plot with trimfill of the meta-analysis showing BASELINE gingival index scores of the FOLLOW-UP brushing exercises analysing the Löe & Silness (1963) Gingival Index and the modification of this index by Löe (1967) for the comparison of dentifrices with BS and NEGATIVE control dentifrices without BS.** Egger’s test shows a non-significant *p*-value (*p*= 0.31).
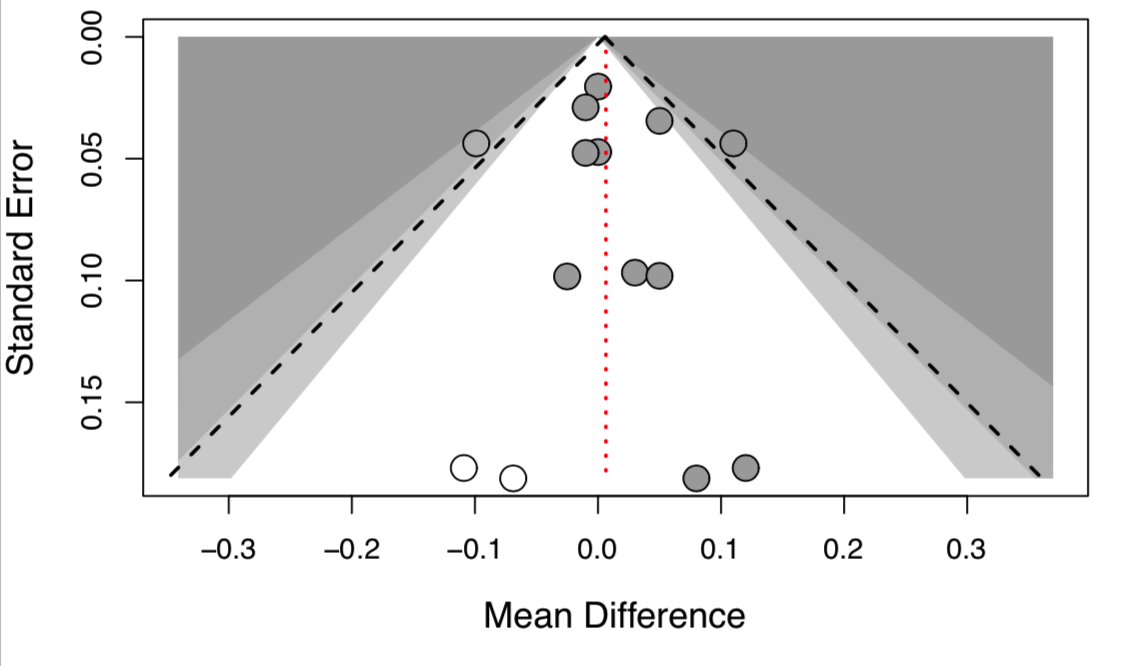


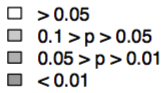


Visually, shows the funnel plot minor asymmetry. However, Egger’s test for asymmetry is not significant (*p*=0.31), indicating a publication bias mechanism is not a major cause for concern here (71). Also, the contour-enhanced funnel plot shows that the “missing” studies are expected to lie in areas of high statistical significance (whereas the majority of available studies are nonsignificant), indicating that the observed asymmetry may not be due to publication bias based on statistical significance (70).

**S10-B) A Contour-enhanced funnel plot with trimfill of the meta-analysis showing END gingival index scores of the FOLLOW-UP brushing exercises analysing the Löe & Silness (1963) Gingival Index and the modification of this index by Löe (1967) for the comparison of dentifrices with BS and NEGATIVE control dentifrices without BS.** Egger’s test shows a significant *p*-value (*p*= 0.009).

Visually, the plot shows asymmetry. Missing studies probably lie within the white non-significant white region and in the significant colored and shaded region (71).

Egger’s test shows a significant *p*-value (*p*= 0.009) leading to rejection of the null hypothesis of symmetry in the funnel plot (73).

**Appendix S11. Network meta-analysis.**

**S11-A) 1.** Network meta-analysis graph for the **Single-brushing** Exercises end scores analysing the Turesky et al. (1970) modification of the Quigley and Hein (1962) **Plaque** Index for the comparison of dentifrices with BS and control dentifrices without BS.


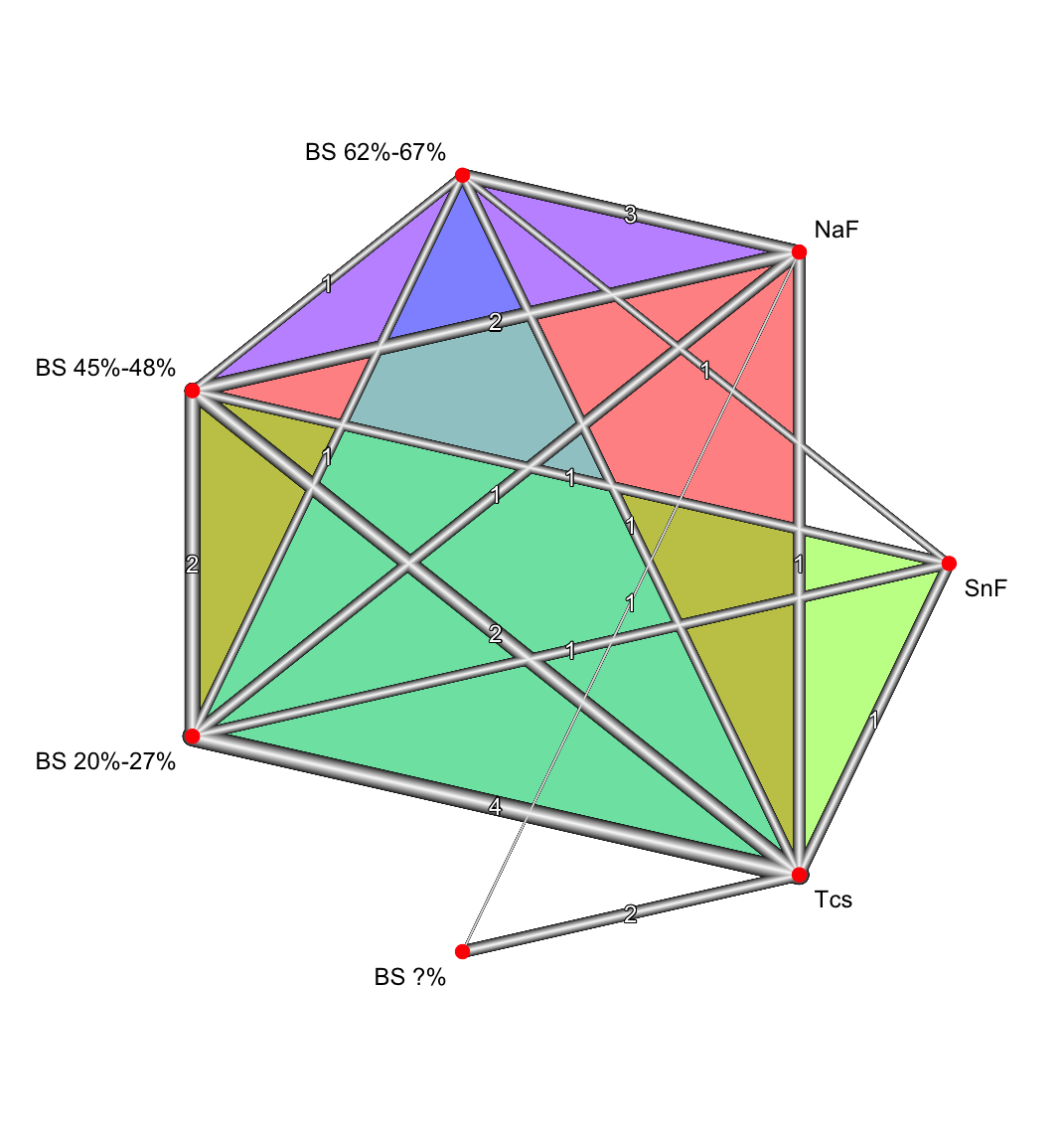


**Studies: 11; treatments: 7; pairwise comparisons: 25.**

The treatments are equally spaced on the perimeter of the circle. Any two treatments are connected by a line when there is at least one study comparing the two treatments. The thickness of the line is proportional to the inverse standard error of the direct treatment comparison. Each comparison may have data from several studies that may be combined in a traditional meta-analysis. The shading indicates the studies with more than two arms. The set of nodes corresponds to the considered treatments and the edges display the treatment comparisons which are directly compared of all included trials.


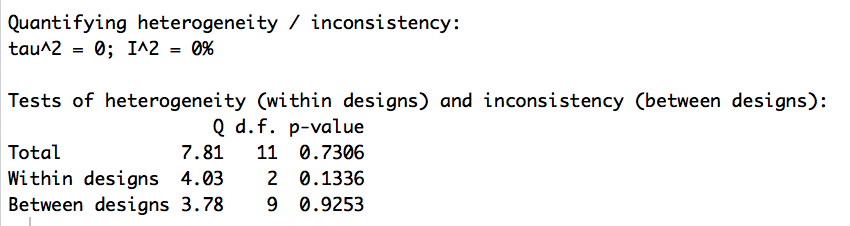


**S11-A) 2. Ranking for the Single-brushing TQ&H End plaque scores**

| **Treatment**  **ranking** | **#**  **Experiments** | **#**  **Participants** | **P-score**  **ranking *** | **MD** | **95%-CI** |
| --- | --- | --- | --- | --- | --- |
| BS unknown % | 3 | 112 | 0.99 | 0.00 |  |
| BS 62%-67% ** | 5 | 271 | 0.83 | 0.11 | (-0.03; 0.27) |
| BS 45%-48% | 3 | 190 | 0.66 | 0.19 | (0.04; 0.33) |
| BS 20%-27% | 4 | 236 | 0.52 | 0.23 | (0.10; 0.37) |
| Tcs | 6 | 338 | 0.22 | 0.34 | (0.22; 0.45) |
| SnF | 2 | 120 | 0.17 | 0.35 | (0.19; 0.50) |
| NaF | 5 | 222 | 0.11 | 0.37 | (0.22; 0.51) |
| **Comparison: Other Treatments vs “BS unknown %”** | | | **Prediction Interval:** | | **(0.05; 0.49)** |

* The frequentist P-scores allow ranking treatments on a continuous 0-1 scale. The P-score of treatment is defined as the mean extent of certainty that treatment is better than another treatment; scale: 0 (worst) to 1 (best). At least under normal assumption the order depends largely on the point estimates ((23).

** data from Mason et al. (2017) and Bosma et al. (2018) are pooled

**Forest plot of the** **Ranking for the Single-brushing TQ&H End plaque scores sorted by ranking.**

**S11-A) 3. Heterogeneity/ inconsistency between designs.**

The *Q*total statistic (of the “whole network”) can be decomposed into a *Q* statistic for assessing the heterogeneity between studies with the same design (“within designs”) and a *Q* statistic for assessing the design inconsistency (“between designs”). Designs are defined by the subset of treatments compared in a study (73).

In the 11 studies included in the network meta-analysis, 9 different designs are used. Since there are only 2 designs for which we have more than two studies, the remaining design specific Q statistics are equal to zero and have no degrees of freedom (31).


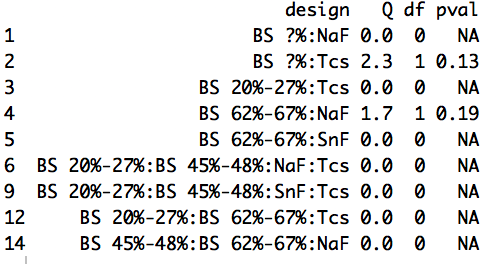


**S11-A) 4. Net Heat Plot** **for detecting inconsistency between of the Single-brushing TQ&H End plaque scores from a random effects model.**

**Inconsistency-detecting heat map function *netheat* from the *netmeta* package applied to the Single-brushing TQ&H End scores data set.** “Hotspots” of inconsistency are absent in the *netheat* plot.

The net heat plot shows a marked reduction in inconsistency.

For a valid analysis of the network, consistent information from different pathways is assumed. Consistency can be checked by contrasting effect estimates from direct comparisons with the evidence of the remaining network. If corresponding treatment effect estimates of various connections, differ between two treatments, there is inconsistency (29).

Treatment comparisons for which there is only one source of evidence are omitted (31).

The area of the gray squares displays the contribution of the direct estimate in design (shown in the column) to the network estimate in design (shown in the row). A clustering procedure is applied to the heat matrix in order to find warm colored hot spots of inconsistency. Blue colors indicate low level of inconsistency, while red colors indicate “hot spots” of high inconsistency (29).

**S11-B) 1.** Network meta-analysis graph for the **Follow-up End** scores analysing the Turesky et al. (1970) modification of the Quigley and Hein (1962) **Plaque** Index for the comparison of dentifrices with BS and control dentifrices without BS.

**Studies: 10; treatments: 6; pairwise comparisons: 14.**

Treatments are represented by nodes and head-to-head studies between treatments are represented by edges.


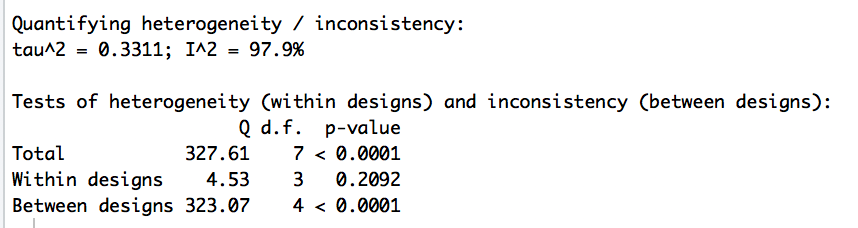


**S11-B) 2. Ranking for the Follow-up TQ&H End plaque scores**

| **Treatment** | **#**  **Experiments** | **#**  **Participants** | **P-score**  **ranking *** | **MD** | **95%-CI** |
| --- | --- | --- | --- | --- | --- |
| Tcs | 3 | 169 | 0.90 | -0.50 | (-1.15; 0.15) |
| MFP | 1 | 20 | 0.64 | -0.16 | (-1.30; 0.98) |
| BS | 10 | 560 | 0.57 | 0.00 |  |
| Non-BS | 3 | 106 | 0.39 | 0.19 | (-0.46; 0.83) |
| NaF | 3 | 255 | 0.32 | 0.28 | (-0.37; 0.93) |
| MFP+NaF | 2 | 80 | 0.18 | 0.49 | (-0.28; 1.26) |
| **Comparison: Other Treatments vs “BS”** | | | **Prediction Interval:** | | **(-0.71; 0.85)** |

* The frequentist P-scores allow ranking treatments on a continuous 0-1 scale. The P-score of treatment is defined as the mean extent of certainty that treatment is better than another treatment; scale: 0 (worst) to 1 (best). At least under normal assumption the order depends largely on the point estimates (23).

**Forest plot of for the Follow-up TQ&H End plaque scores sorted by ranking *.**

* The frequentist P-scores allow ranking treatments on a continuous 0-1 scale. The P-score of treatment is defined as the mean extent of certainty that treatment is better than another treatment; scale: 0 (worst) to 1 (best). At least under normal assumption the order depends largely on the point estimates (23).

**S11-B) 3. Heterogeneity/ inconsistency between designs.**

The *Q*total statistic (of the “whole network”) can be decomposed into a *Q* statistic for assessing the heterogeneity between studies with the same design (“within designs”) and a *Q* statistic for assessing the design inconsistency (“between designs”). Designs are defined by the subset of treatments compared in a study (73).

In the 10 studies included in the network meta-analysis, 8 different designs are used. Since there are only 3 designs for which we have more than two studies, the remaining design specific Q statistics are equal to zero and have no degrees of freedom. Except for design BS: Tcs (p=0.521) and BS: NaF (p=0.768) , for the other design BS: Non-BS (p=0.045) there is more heterogeneity between the contributing studies than we would expect by chance (31).

**The total within-design heterogeneity further decomposed into the contribution from each design:**

**
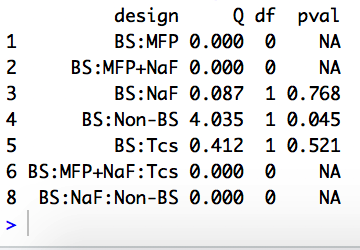
**

**S11-B) 4. Net heat plot for detecting inconsistency between designs of the Follow-up TQ&H End plaque scores from a random effects model.**

**A heat map plot for the detection of inconsistency (73).** Several “hotspots” of inconsistency can be seen between the effects in designs.

**S11-C) 1.** Network meta-analysis graph for the **Follow-up End** scores analysing the Löe & Silness (1963) and Löe (1967) **Gingival** Index for the comparison of dentifrices with BS and control dentifrices without BS.


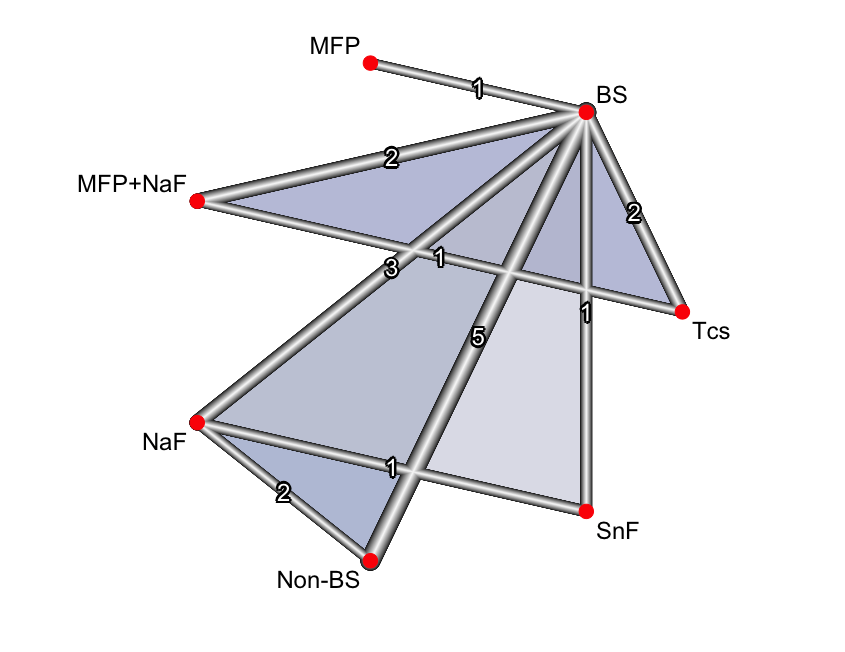


**Studies: 10; treatments: 7; pairwise comparisons: 18**

Treatments are represented by nodes and head-to-head studies between treatments are represented by edges.


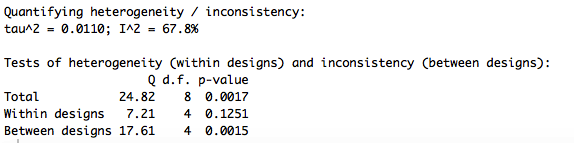


**S11-C) 2. Ranking for the Follow-up End Scores according the Löe & Silness Gingival Index.**

| **Treatment**  **ranking** | **#**  **Experiments** | **#**  **Participants** | **P-score**  **ranking *** | **MD** | **95%-CI** |
| --- | --- | --- | --- | --- | --- |
| Tcs | 2 | 67 | 1.00 | **-0.36** | **(-0.54; -0.19)** |
| SnF | 1 | 267 | 0.72 | 0.11 | (-0.28; 0.11) |
| Non-BS | 5 | 143 | 0.60 | 0.19 | (-0.15; 0.10) |
| BS | 10 | 409 | 0.53 | 0.00 |  |
| NaF | 3 | 176 | 0.28 | 0.07 | (-0.08; 0.22) |
| MFP | 1 | 20 | 0.22 | 0.35 | (-0.12; 0.36) |
| MFP+NaF | 2 | 80 | 0.16 | 0.37 | (-0.03; 0.28) |
| Comparison: Other Treatments vs “BS” | | | **Prediction Interval:** | | **(-0.50; 0.44)** |

**Forest plot of for the Follow-up End scores according the Löe & Silness Gingival Index.**


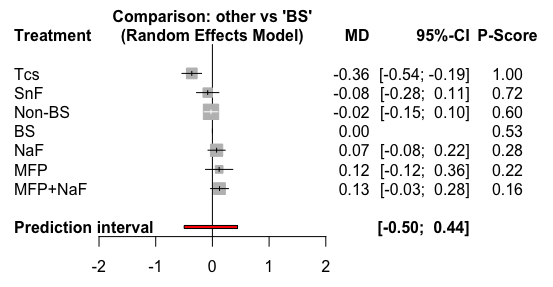


* The frequentist P-scores allow ranking treatments on a continuous 0-1 scale. The P-score of treatment is defined as the mean extent of certainty that treatment is better than another treatment; scale: 0 (worst) to 1 (best). At least under normal assumption the order depends largely on the point estimates (23).

**S11-C) 3. Heterogeneity/ inconsistency between designs.**

In the 10 studies included in the network meta-analysis, 9 different designs are used. Since there are only two designs for which we have more than two studies, the remaining design specific Q statistics are equal to zero and have no degrees of freedom. Except for design BS: Non-BS (p=0.954), for the other design BS: NaF: Non-BS (*p*=0.028) there is more heterogeneity between the contributing studies than we would expect by chance (31).

**The total within-design heterogeneity further decomposed into the contribution from each design:**


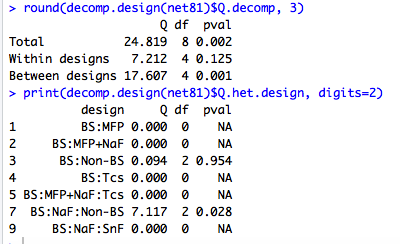


**S11-C) 4. Net heat plot for detecting inconsistency between designs of the Follow-up Löe & Silness End gingival index scores from a random effects model.**


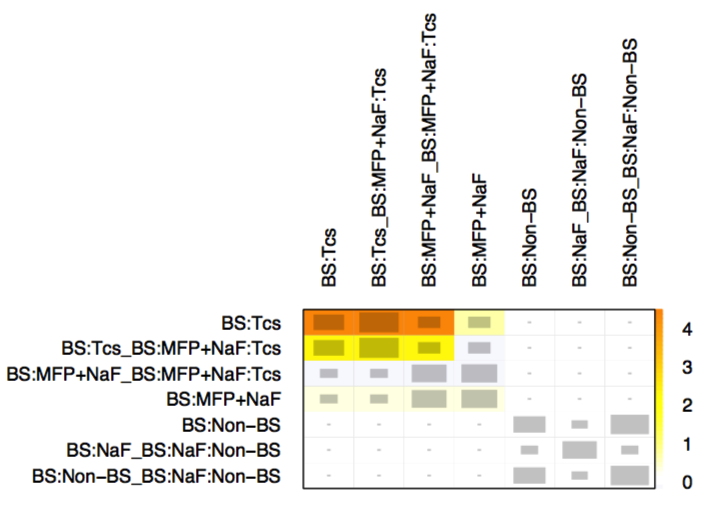


**A heat map plot for the detection of inconsistency.** Warm orange colors indicate inconsistency of evidence.
